# Enhancing mechanical attributes and tribological performance of titanium friction stir welded joints through nanoparticle reinforcement

**DOI:** 10.1038/s41598-025-18558-x

**Published:** 2025-10-06

**Authors:** K. Giridharan, A. Praveen Kumar, G. Chakravarthi, R M. Sakthi Sadhasivam, D. Manikandan, Krishnaraj Ramaswamy

**Affiliations:** 1 Department of Mechanical Engineering, Easwari Engineering College, Chennai, Tamilnadu India; 2 Department of Production Engineering, NIT, Tiruchirappalli, Tamilnadu India; 3 Department of Mechanical Engineering, SRM TRP Engineering College, Tiruchirappalli, Tamilnadu India; 4 Department of Mechanical Engineering, College of Engineering and Technology, Dambi Dollo University, Dembi Dolo, Ethiopia; 5 Center for Global Health Research, Saveetha Institute of Medical and Technical Sciences, Saveetha University, Chennai, Tamilnadu India

**Keywords:** Friction stir welding, Titanium grade 4, Biochar, Mechanical characteristics, Microstructure properties, Wear resistance, Engineering, Mechanical engineering

## Abstract

In recent decades, friction stir welding has emerged as a significant transformative technique in advanced manufacturing. In this work, fabricating the friction stir welding of 5 mm thick similar titanium grade 4 plates with varying weight percentages of naturally derived biochar (0, 1, 2, and 3 wt%) to enhance the mechanical properties and wear behavior. According to the test analysis, the biochar-included FSW samples had better mechanical properties than the plain-welded titanium sample. The 2 wt% biochar-induced FSW sample gets the best tensile result of 395 MPa, impact strength of 32.02 J, and fatigue result of 183 MPa. Through field emission scanning electron microscopy analysis, biochar was well dispersed throughout the regions and contributing to the grain nucleation. The bookend surfaces of the tensile fractured specimen were examined by using scanning electron microscopy analysis. According to micro-hardness and wear resistance test analysis, a 3 wt% biochar sample plays a key role in enhancing the results and getting 107 HV and a specific wear rate of 0.052 mm³/Nm×10^− 3^ and a co-efficient of friction of 0.25 µ. The worn surface analysis was accomplished by scanning electron microscope analysis, and the wear mechanisms were studied. The novel approach of the present research is to suggest the ideal biochar FSW titanium sample for aerospace components in critical wear applications. This technique is currently gaining popularity and is being used in a variety of applications, including aviation, shipbuilding, aircraft companies, defense sectors, and the automotive industry. These are implemented to mitigate the detrimental effects and the emergence of defects in the joining of similar alloys in comparison to fusion welding techniques because of their energy-efficient, versatile, and eco-friendly process.

## Introduction

The innovative joining technique named Friction Stir Welding was developed in 1991 and used primarily for joining metals without reaching their melting point. It involves a rotating tool that generates frictional heat and makes the material soften, enabling the mixing of materials to form a solid-phase weld^1–3^. FSW is chiefly advantageous for joining high-strength alloys and dissimilar materials because it avoids the defects commonly accompanying conventional fusion welding methods, namely distortion, porosity, reduced residual stresses, and cracking. The process results in fine-grained microstructures in the weld zone region, and the results extract to enhance the mechanical aspects of the weld joints^4,5^. Moreover, its low heat contribution also minimizes thermal stresses, which makes it favorable for welding temperature-sensitive materials. FSW is particularly beneficial for joining metals like high-strength alloys, aluminium, and magnesium. Because of these advantages, FSW is extensively used in railway, automotive, shipbuilding, and aerospace industries^6–8^.

Titanium (Ti) is a high-performance metal widely used in industries demanding greater strength, low density, and excellent corrosion resistance^9^. These exceptional mechanical properties make titanium a critical metal in chemical processing, aerospace, marine, and biomedical industries. Generally, the high strength-to-weight ratio and biocompatibility allow its use in structural components like aircraft frames, marine structures, and medical implants and extend to utilize in severe atmospheric conditions. Moreover, its high resistance to extreme temperatures and corrosive mediums delivers a significant advantage in demanding applications, ensuring reliability and durability over a longer lifetime^10–12^.

Among the various titanium alloys, titanium Grade 4, commonly known as commercial pure titanium (CP-Ti Grade 4), is the toughest material among the unalloyed titanium grades. It offers a good balance between strength, ductility, and corrosion resistance. This grade is commonly used in FSW due to its high strength and compatibility with the solid-state process and prevents the formation of brittle phases that may occur in conventional welding processes^13–15^. Its use in industries like aerospace, chemical processing, and the medical field highlights their capability and versatility to meet strict performance standards. Also, the ability to withstand high-stress environments and its biocompatibility make titanium grade 4 an ideal candidate for FSW applications where both mechanical performance and long-term durability are vital^16^.

Friction stir processing (FSP) is an extension of FSW used to improve the properties of metals through confined microstructural modification. The FSW attention on joining and FSP is focused on refining grain structures, enhancing mechanical behavior, and improving surface wear resistance^17,18^. By applying the similar principles of plastic deformation and dynamic recrystallization as FSW, FSP enables effective control over material properties such as fatigue resistance, toughness, and hardness. FSP is specifically beneficial in improving surface properties of the weld joints without affecting the parent material and making it favorable in different sectors, like aerospace industries and automotive fields, where surface integrity is significant^19,20^.

Biochar is a carbon-rich material derived from biomass, commonly rice husk. It is emerging as an advantageous reinforcement in metal matrix composites due to its lightweight, high thermal stability, and superior lubrication behavior^56,60^, with properties comparable to other natural reinforcements like date seed fillers. When biochar is used as a reinforcing particle in FSP, it correspondingly enhances wear resistance, reduces friction, and improves thermal stability by forming a protective tribolayer during mechanical loading^21^. The current ceramic reinforcements, such as silicon carbide, alumina, boron carbide, graphite, zinc oxide, etc., are broadly used in industrial applications with metal matrix^22,23^. But biochar was chosen due to its renewable origin, low density, and porous carbon structure that promotes stable tribolayer formation during sliding. Unlike SiC or B₄C, biochar produces minimal interfacial reactions with titanium and preserves matrix integrity under tribological loading. Also, its lubricating nature makes it effective for wear resistance compared to traditional inorganic reinforcements^24^. The inclusion of biochar in FSP processes significantly improves the mechanical and tribological properties of metals like titanium, similar to enhancements observed in aluminum matrix composites^57^, and offers a sustainable approach to enhancing material performance in high-demand applications.

Sina Alipour et al. worked on fabricating polypropylene (PP)/acrylonitrile butadiene rubber (NBR) composites reinforced with halloysite nanotubes (HNTs) and dual compatibilizers (PP-g-MA and SEBS-g-MA) with varied NBR (10–30 wt%) and HNTs (3–7 wt%) concentrations. FSW of 80/20 (wt/wt) PP/NBR composites with dual compatibilizers significantly reduced NBR droplet size and improved dispersion of nanoparticle, improving weld joint properties due to the stirring action of the FSW tool^25^. Rajesh et al. work examines the electron beam welding of AA2024 aluminium alloy by controlling heat input and avoiding defects typically associated with conventional welding methods. The welded joint demonstrated a tensile strength of 285 MPa, equivalent to 62% of the base material strength, owing to refined grains at the weld interface. Additional strengthening was achieved through copper aluminide precipitate formation in the weld zone^26^. Sivashanmugam et al. detailed a study on the joining of magnesium (ZE41) and a similar alloy and evaluated the mechanical and corrosion behavior suitable for biomedical applications through the friction stir welding process. The study established the development of a defect-free weld with a tensile strength efficiency of 79.25% and hardness variations across the weld zones.

Micro-arc oxidation coatings were applied to enhance the corrosion resistance and exhibit significantly lower corrosion rates of 12.45 mpy due to the formation of a protective oxide layer and apatite deposition. Therefore, this property makes the material suitable for biomedical applications^27^. Deepak Kumar et al. combines experimental and computational fluid dynamics (CFD) modeling to analyse heat flux, temperature distribution, strain rate, and viscosity, validated by experimental temperature data of AA2024-T4. Results reveal symmetric heat flux and strain rate about the tool axis but asymmetric material flow influencing grain size and mechanical properties^28^. Abolfazl Khalkhali et al. employ Taguchi-based design of experiments and ANOVA to analyse the impact on forces, temperature, tensile strength, elongation, grain size, hardness, and weld zone thickness in AA1100 alloy. A perceptron neural network and multi-objective optimization (NSGA-II, NIP, TOPSIS) were used to optimize pin profiles and achieve minimal forces (1452 N horizontal, 2913 N vertical), fine grain size (2 μm), high hardness (57.2 HV), tensile strength (2126 N), and temperature (374 °C). These findings align with prior FSW studies, demonstrating the process capability to enhance joint quality and performance^29^.

Appasaheb N Pandav et al. study employs Principal Component Analysis (PCA) and Taguchi-Grey Relational Analysis (GRA) to optimize FSW process parameters, including traverse speed, rotational speed, tool tilt angle, shoulder diameter, and tool pin profile. Using a Taguchi L16 orthogonal array, experiments assessed tensile strength, yield strength, elongation, hardness, and bending load, identifying optimal traverse speed at 160 mm/min, rotational speed at 900 rpm, tilt angle at 2°, shoulder diameter at 16 mm, and straight square pin profile. ANOVA revealed rotational speed as the most influential factor (43.56%), with confirmation tests showing a 19.06% improvement in weld joint quality^30^. Giridharan et al. concentrated on optimizing friction stir welding process parameters to improve the mechanical behavior of dissimilar metal welds of copper and steel. Research has exposed that axial force, welding speed, and tool rotational speed significantly influence welding strength. Using Taguchi’s method and L9 orthogonal arrays identified ideal input conditions for maximized mechanical properties. The studies mainly focused on the importance of precise control over the input parameters and achieving defect-free joints with good surface integrity^31^. Vetrivel Sezhian et al. explored the improvement of mechanical properties in copper butt joints reinforced with B_4_C nanoparticles using FSW. By reinforcing B_4_C (1–4 wt%), there is a significant improvement of the tensile strength, fatigue strength, and microhardness of the weld. Among the samples, 3 wt% B_4_C attained the highest results of tensile (203 MPa), fatigue (159 MPa), and hardness (123 HV), respectively. Microstructural analysis exposed uniform dispersion of B_4_C particles and aids in grain refinement. This enhanced the weld properties through improved thermal conductivity and lubrication during FSW. The findings also suggest that B_4_C reinforcement is an effective technique for generating strong and defect-free welds^32^. Giridharan et al. experimented with low-carbon steel AISI-SAE 1010 and copper CDA 101 by friction stir welding with a tapered pin tool at 900 rpm stirring speed, 30 mm/min traverse rate, and 5 kN axial force. Fine ferrite grains in the stir zone due to dynamic recrystallization were revealed from microstructural analysis and result in high hardness and mechanical properties. The tensile strength of 181.5 MPa, elongation of 14.03%, and hardness of 144 VHN were attained^33^.

Most of the studies predominantly focused on friction stir welding of similar and dissimilar metal plates and found their mechanical and microstructural properties. The research related to the FSW of plates along with the inclusion of reinforcement particles was limited. The aim of this research is to propose the ideal weld from the significant investigation to the aerospace component applications, where FSW of titanium was much required. The principal objective of the work is to investigate the microstructural interrelationships, mechanical properties, and wear characteristics of similar titanium (Ti Gr-4) plates along with biochar inclusions by varying the weight% (1, 2, and 3%) fabricated through friction stir welding. This research seeks to extend the understanding about the weld metal microstructure, tensile strength, impact, micro-hardness, and fatigue strength of friction stir welded biochar-included titanium plates.

## Materials and methods

In this research, Titanium Grade 4 material was used as the parent material in its non-heated form for the process. The solid-state base material was procured from Krishna Materials, Chennai. This study uses a 5 mm thick titanium plate with the dimensions of 100 × 50 mm. The quantity of reinforced biochar naoparticles utilized is illustrated in Table 1. The chemical composition and mechanical characteristics of as-received titanium (grade 4) material was presented in Table 2 and The detailed chemical composition of titanium grade 4 is presented in Table 3. The plate was processed by a metal cut-off saw machine, and the rough edges were sized by milling. The rough corners were removed from the base metal by using a pedestal grinding machine. The processed metal was held by manual top clamps in order to perform the welding process. The size and density of the biochar nanoparticle are 100 ± 20 nm with a density of 0.45 ± 0.05 g/cc. They were purchased from Metro Composites, Chennai in a chemically processed state. Usually by using a drilling process, the biochar was incorporated in the base metal at the joining region. But in this research, in order to achieve the precise joining, a wire-cut electrical discharge machine was employed to generate the holes.

Biochar is introduced into pre-drilled cylindrical holes along the abutting surfaces of the plates before welding, allowing them to be incorporated into the weld zone during the FSW process. This innovative approach aims to improve weld properties, microstructure, and mechanical performance. A sequence of 20 plain cylindrical holes are fabricated at a distance of 5 mm per plain plate with the dimensions of 1 mm diameter and 1.5 mm depth, and they were made in the abutting edges of the workpiece by the EDM process. The biochar is stored in these holes, which ensures that they are dispersed in a regulated manner during the FSW process. It is anticipated that the incorporation of biochars will have an effect on a number of significantly important aspects of the welding process and joint qualities, including mechanical and microstructural characteristics. Ball milling is a method that can be used to produce nanoparticles of biochar. A suspension of biochar and ethanol is utilized in order to fill the cylindrical holes in a homogeneous manner. As a result of the ethanol being evaporated, a layer of biochar nanoparticles that has been compressed is left behind within the holes. Initially, as the pinless spinning FSW tool pin moves over the weld line, the strong plastic deformation and heat force the biochar to scatter within the stir zone. This ensures that the nanoparticles do not escape throughout the joining process. In the stir zone, ultrafine grains are produced when biochar serves as nucleation sites for grain refining. As a result of their effect on the FSW dynamic recrystallization mechanism, carbonaceous particles enhance mechanical toughness and strength. The amount of biochar that is integrated into the weld nugget zone is determined by the process of calculating the product of the entire mass of the base material and the weight fraction of the nanomaterial, which are both taken into consideration. The amount of biochar that is integrated into the weld nugget zone is determined by the process of calculating the product of the entire mass of the base material and the weight fraction of the nanomaterial, which are both taken into consideration as shown in equation number 1.

1 $$\:Quantity\:of\:nanopartciles=\frac{1}{100}\times\:\:M$$

where, M -Mass of the base plate in grams.

**Table 1:** Quantity of reinforced nano particle.

Mass of the base material in grams (g)	Nano particle reinforcement	
300	**Wt. %**	**Grams (g)**
	0 (Plain Weld)	-
	1	3
	2	6
	3	9

According to the studies, the tool geometry, including proportions and tool pin profile, provides a major impact on the welding process. So, considering the aspects, a non-consumable form of high-speed steel (grade: H13) was engaged for the work. The major criteria for the choice of the H13 tool are its high strength and its economical nature. The dimension of the tape pin tool maintained is a 25 mm diameter shoulder, a 4.85 mm pin length, and a flute diameter ranging from 6 mm to 2 mm.

**Table 2:** Chemical composition of titanium grade 4.

Element	Fe	O	C	*N*	H	Ti
Weight%	0.5	0.4	0.1	0.05	0.015	Remaining

**Table 3:** Mechanical properties of titanium grade 4.

Mechanical Properties	Ultimate tensile strength (MPa)	Yield strength (MPa)	% of elongation	Hardness (Brinell)
Titanium grade 4	550	480	15	265

### Preparation of biochar nanoparticles

**Fig. 1:**
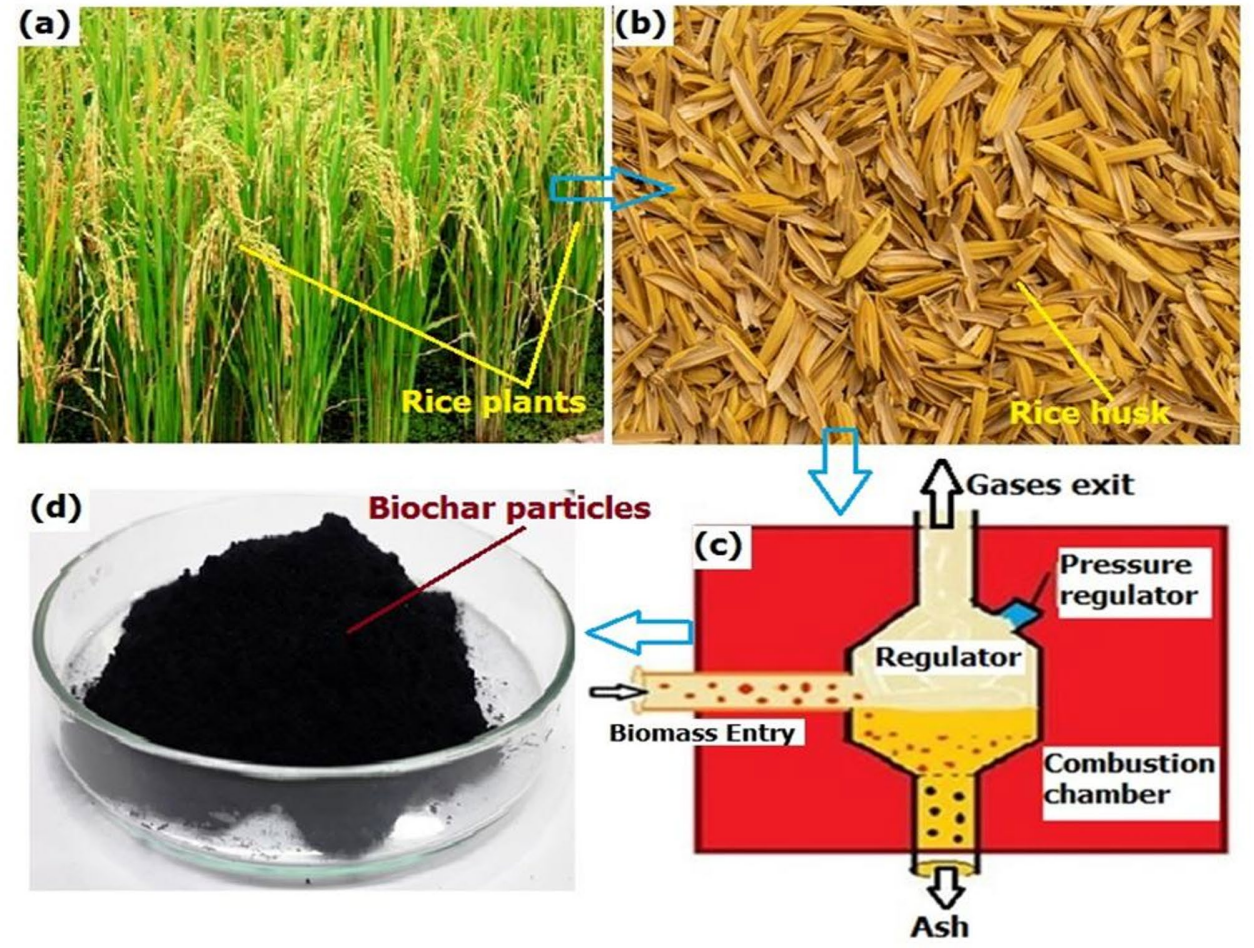
Processing of biochar.

**Fig. 2:**
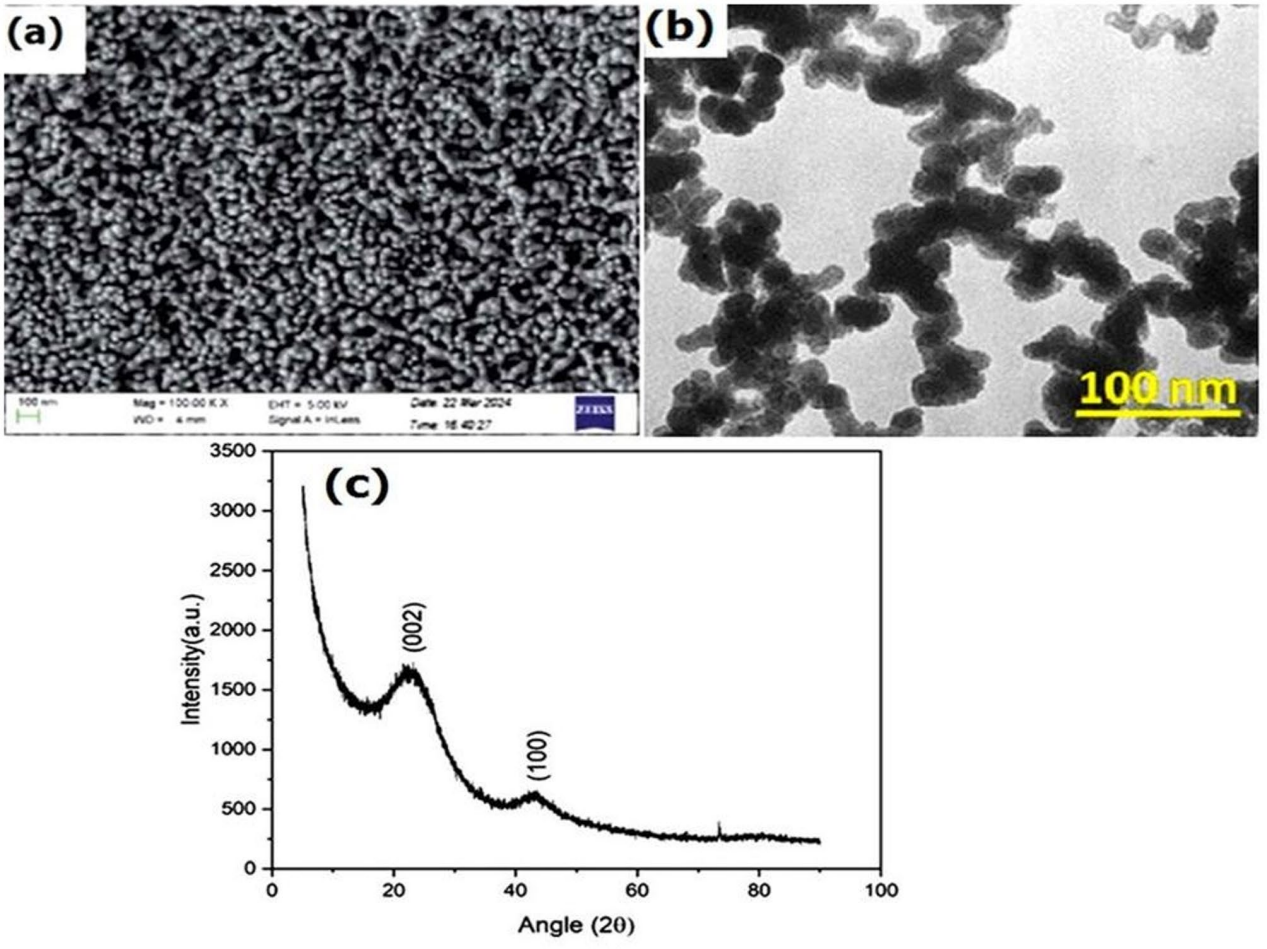
Processed biochar nanoparticle (**a**) SEM, (**b**) TEM Morphology, (**c**) XRD plot.

The rice husk underwent a combustion process and turned into a biomaterial called biochar, which is rich in carbon. This process of converting the agricultural wastes, fibers, crops, and industrial wastes by heating to the temperature of more than 400℃ in the absence of an oxygen environment is called pyrolysis. By following the process, the biochar was made from rice husk material. During the processing, two different forms of components are formed, namely rice husk ash (RHA) and biochar (RHB). Generally, RHA has comparatively low strength and is formed in an oxygen environment. But RHB is rich in carbon and high in strength because it was processed in the absence of oxygen. The biochar is a good replacement for inorganic fibers and fillers due to their low density and biodegradable nature. Though a variety of processing techniques, namely pyrolysis, gasification, carbonization, liquefaction, and combustion are used to convert 27husk to biochar. Carbonization and pyrolysis are the efficient ways for the thermal decomposition of rice husk as biochar. However, the carbonization process is accomplished by utilizing a lower temperature for a longer duration. So, by pyrolysis thermal decomposition, the rice husk was converted into biochar in an inert environment condition. The variety of factors, namely heating rate, pyrolysis temperature, and time period during the pyrolysis, has a direct effect on biochar strength. For the present research, the biochar has a density of 0.45 ± 0.05 g/cc with a diameter of 100 ± 20 nm. The chemical composition of the as-received biochar used in the study was presented in Table 4 and the processing steps of the biochar from rice husk were shown in Fig. 1 (a-d). The SEM and TEM images of the processed biochar are shown in Fig. 2 (a & b). The XRD plot analysis from Fig. 2 (c) indicates that the XRD pattern of the biochar matches the crystalline structure of carbon. It is characterized by the hexagonal crystal structure and typically shows prominent peaks at specific 2θ angles at 26.5° ((002) plane), and it is an indication of layered structure.

**Table 4:** Chemical composition of as received biochar.

Element	C/*N*	H/C	*N*	H	C
Weight%	54.90	0.095	0.685	3.61	40.71

### Friction stir welding process

**Fig. 3:**
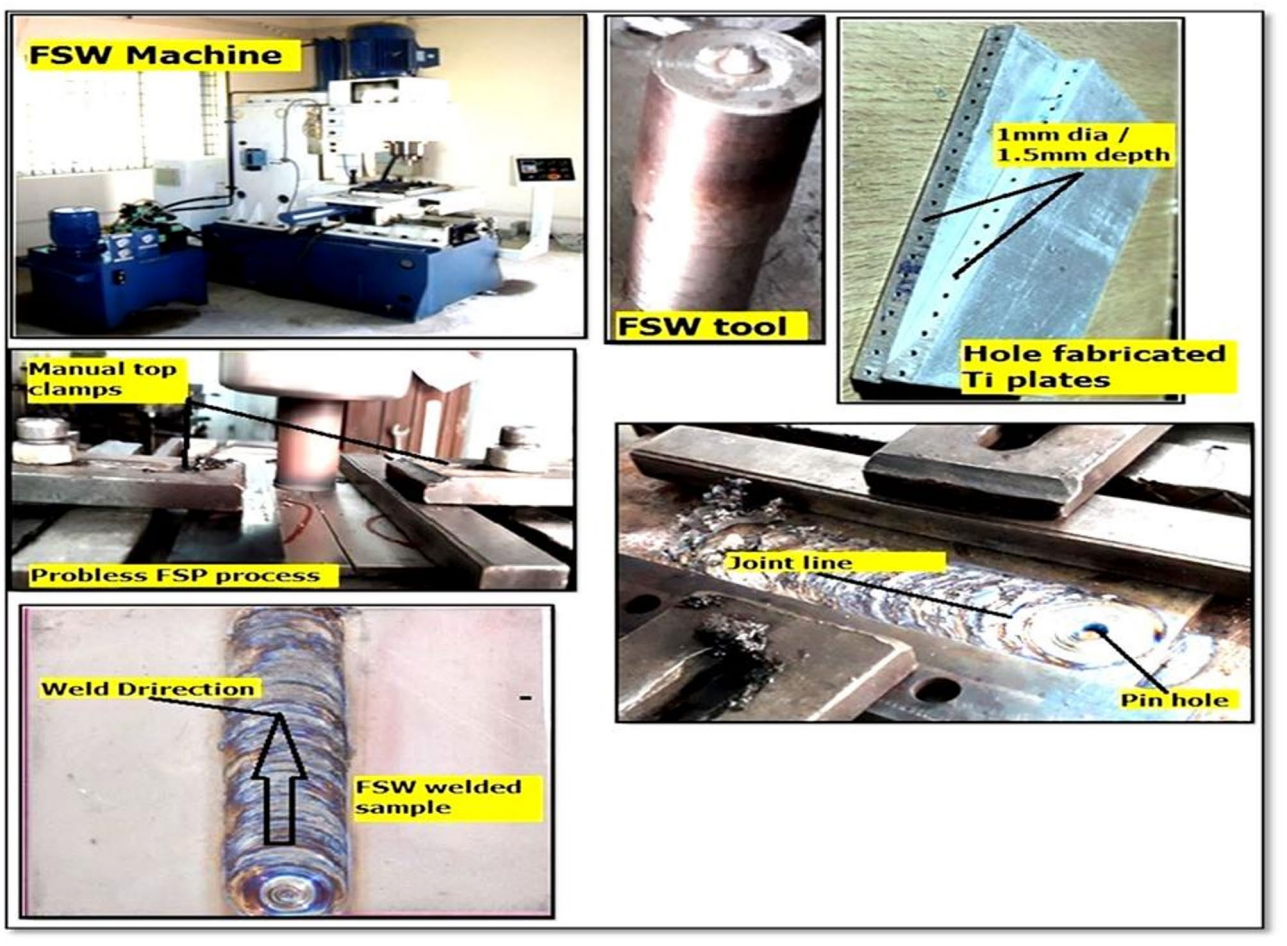
Photographic view of Friction stir welding process.

In this study, friction stir welding (FSW) was performed with biochar reinforcement using a vertical milling machine having an FSW setup. The specification of the FSW machine is 5 kW motor power capacity, 820 mm length of machine bed, and movement of 40 mm width. The experiment was executed at S.A. Engineering College, Chennai, and the setup along with the tool, workpiece, and tested samples was shown in Fig. 3.

The parent material employed for the work was titanium grade 4 alloy. So, on both the advancing and retreating sides, the same processed titanium workpiece was placed with the proper assistance of clamps. The workpiece on the advancing side was first advanced into the weld and highly deformed. It forms an arc-shaped structure when viewed down the tool axis, and some layers are broken up into tiny fragments. When the tool moves in the region of the retreating side, the lighter materials pulled out are dragged around to the rear side of the tool and occupy the gaps between the arcs. The entire detached material did not move around the pin and resulted in a larger grain size with minimal deformation. Primarily, the base metal on the advancing side has attained higher tensile strength, yield strength, elongation percentage, and hardness results. On the advancing side, the amount of heat produced is more because higher friction is developed in the regions. This developed heat will produce the proper weld with the retreating side of the alloy. Then by the friction stir processing method, abutting surface geometry has been closed by biochar using the tool with a 20 mm diameter rotating at 360 rpm speed. This process was specifically used for the reinforcement of biochar into the weld region. A tapered cylindrical pin profile was selected to promote effective material stirring and uniform distribution of biochar particles. A plunge depth of 0.5 mm and a dwell time of 5 s ensured adequate heat generation and material consolidation without excessive flash formation. Also, the controlled heat input was critical for enhancing material flow and preventing void formation during nanoparticle incorporation. The tool revolved from one end of the weld to the other in the transverse direction by maintaining the proper boundaries without any deviations. The input parameters and their ranges used for the reinforcement of biochar in the weld were presented in Table 5.

**Table 5:** Details of process parameters.

Welding parameters	Input type and ranges
Biochar nanoparticle	0, 1, 2, and 3 wt%
Dwell time	5 s
Plunge depth	0.5 mm
Welding joint length	100 mm
Axial force	5 kN
Tool pin profile	Taper cylindrical
Welding speed	40 mm/min
Tool rotational speed	1400 rpm

**Fig. 4:**
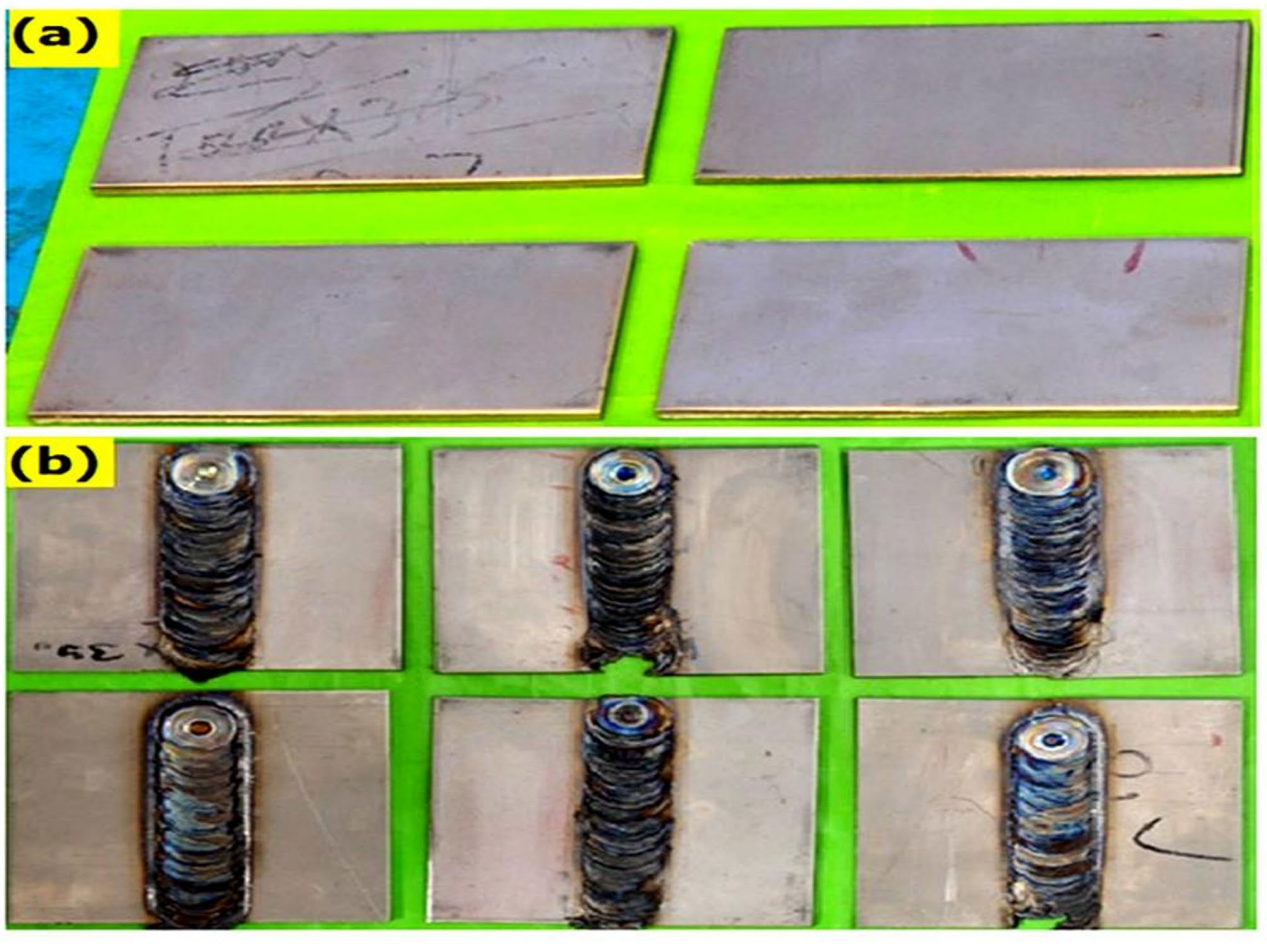
Photographic view of (**a**) Before weld (**b**) After weld samples.

By using a similar procedure, the welding joints were made along with three different reinforcements of biochar (0, 1, 2, and 3 wt%) and designated as samples 1, 2, 3, and 4. The visual examination was made to analyse the weld regions to examine if they are free of defects, and the morphological characterizations were performed on the specimen. The photographic view of before and after Titanium grade 4 weld samples is shown in Fig. 4 (a & b).

The microstructure of the weld was analysed by using a De-wintor inverted trinocular metallurgical microscope. Further, the profound investigation of weld characterization by FEI Quanta FESEM. The test sample preparation for microstructure characterization was performed by grinding the mounted sample with a series of emery papers of various grit sizes (120 to 2000). Following the rubbing, the specimen was polished using a buffing machine by applying the silicon carbide paste. For observing the fine grains, the Kroll’s Reagent etchant solution was applied on the surface of the prepared specimen.

Along with the microstructural characterization through FESEM, the elemental composition by energy dispersive spectroscopy was also made. The strength of the biochar-included FSW specimen was investigated by tensile, impact, and hardness tests. The tensile test samples were prepared as per the ASTM E8M-04 standard, and tests were made by the FIE Universal Testing Machine (UTM), which has the loading capacity of 40 tons with a crosshead speed of 2.5 mm/min. The impact test of the samples was proceeded by the FIE impact tester as per the ASTM E23 standard and had a capacity of 50 J. For both the tensile and impact tests, three specimens were prepared from each sample, and the average was used for the precise analysis. Then the hardness test was performed on the sample by the Wilson Wolpert Vickers micro-hardness tester by following the standard of ASTM 384. For each sample, five indentations were made randomly on the weld regions, and the average was used. The wear test was made on the specimen, and their resistance to wear was evaluated by using a pin-on-disc apparatus as per ASTM G99-95 standard. The test was made against EN31 oil-hardened steel, and the process inputs were maintained at a constant sliding velocity of 3 m/s, a sliding distance of 1000 m, and an applied load of 30 N, respectively. The worn surface of the wear-tested samples is investigated through Scanning Electron Microscope (APREOS) and the morphological analysis is performed.

### Fatigue test

The visually examined titanium grade 4 welded components were cut in the appropriate manner by applying a wire cut electrical discharge machining method in accordance with ASTM E466-07. The distilled water was used as the dielectric medium, and the wire diameter was 0.25 millimeters. The standoff distance was 0.4 millimeters, the pulse width was 113 volts on time and 25 volts off time, the current density was 230 volts, and the gap voltage was 24 volts, used as input parameters while preparing fatigue test specimens. Fatigue testing is an essential technique in the fields of material science and mechanical engineering that is utilized to gain an understanding of how materials respond when subjected to repeated or variable loads over a period of time. It is able to make accurate predictions regarding the lifespan of a material or component when it is subjected to cyclic stress. For the purpose of providing a thorough understanding of the FSW weld samples during the fatigue test process, the conditions play a significant role in evaluating the fatigue strength of the weld samples. These variables include the type of load that is applied, the number of cycles, the preparation of the sample, and the testing techniques. The uniaxial tensile load is the sort of loading that is utilized in fatigue testing the most frequently. In this type of loading, the material is pulled mostly along one axis. Depending on the configuration of the test, the material is subjected to alternating tension and compression, or sometimes they are subjected to pure tension. A tensile-tensile fatigue tester manufactured by MTS Load Frame, United States was utilized in order to evaluate the fatigue strength of a number of specimens that had been welded. For the duration of the test, the settings were maintained at 10 Hz, 0.1 stress ratio, and 23 degrees Celsius for the operating temperature. The fatigue strength was determined by performing 10^7^ cycles at a stress level that was 50% of the ultimate tensile stress of the test specimen.

## Result and discussion

### Microstructure studies of weld joint

**Fig. 5:**
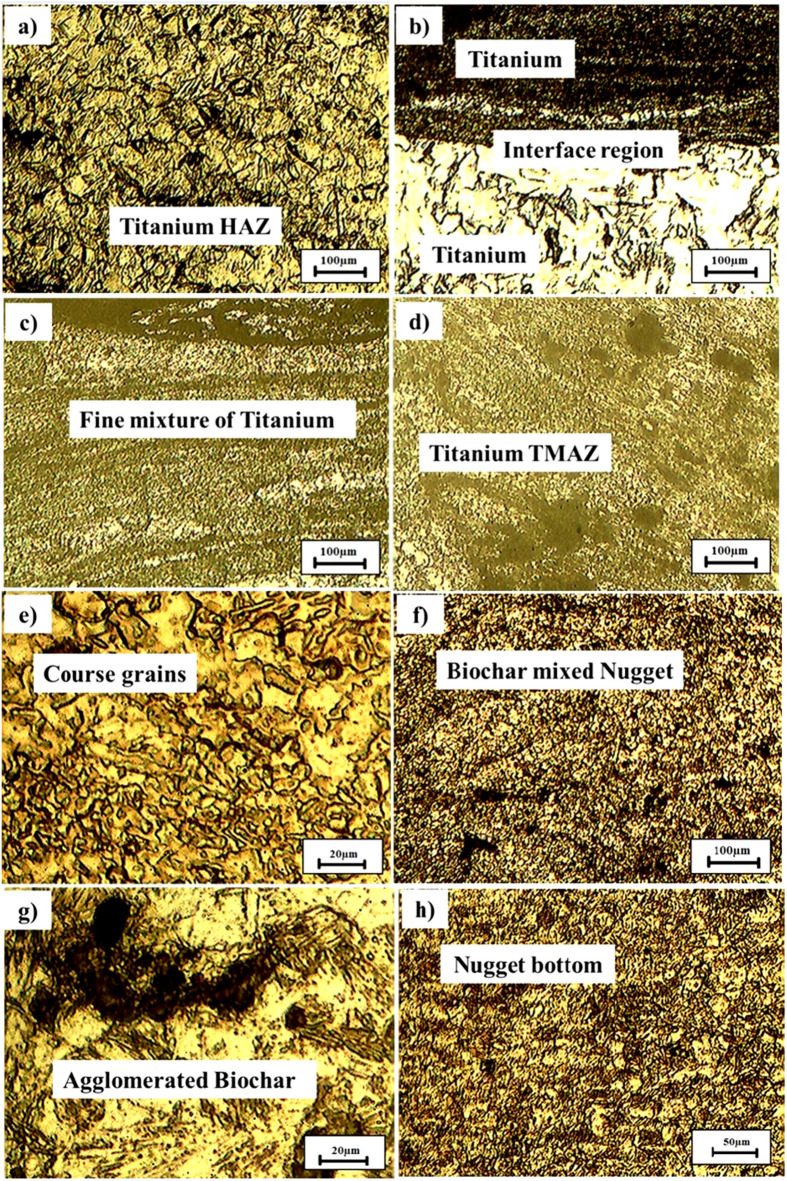
Microstructure of the plain weld (**a-b**), 1 wt% biochar weld (**c-d**), 2 wt% biochar weld (**e-f**), and 3 wt% biochar weld (**g-h**).

The microstructural studies involve the FSW of titanium alloy plates with the inclusion of varied concentrations of biochar by metallurgical microscope, and the micrographs are shown in Fig. 5 (a-h). The micrographs are taken in the regions of the heat affected zone (HAZ), thermomechanical heat affected zone (TMAZ), biochar -induced area, and interface regions (weld nugget). The sample without biochar exhibited the typical characteristics of FSW, such as HAZ, TMAZ, and the stir zone. From Fig. 5 (a-b), it was analysed that the stir zone appeared fine with equiaxed grains. This was due to the dynamic recrystallization attained by the plastic deformation of titanium during welding. However, particularly at the interface of TMAZ and the stir zone, some of the minor cracks, such as microvoids and surface roughness, were observed^34,35^. Continuously, the FSW with 1 wt% biochar inclusion sample was analysed and shown in Fig. 5 (c-d).

From the analysis, it was noticed that slightly rougher textures were attained due to the interactions of titanium and biochar. Also, the homogeneous distribution of biochar was found with tiny clusters. So, the nanoparticles serve as nucleation sites for grain refinement in the stir zone^36,37^. The homogeneous dispersion of biochar within the matrix further contributes to a more consistent microstructure, as evidenced in research on carbon-based additives in FSW composites^38^. But the surface roughness was marginally higher than sample 1, without the indication of major defects such as cracks and voids. Following that, the weld sample 3 with 2 wt% biochar inclusion was studied, and the result was shown in Fig. 5 (e-f). The stir zone of the sample exhibits more influence of biochar than samples 1 and 2. From Fig. 5 (e-f), it was inferred that biochar was densely distributed and leads to increased grain refinement. The surface had appeared smoother in biochar-distributed regions and indicates the accomplishment of proper material flow during the FSW process. The presence of some biochar agglomerations leading to the microvoid formation at the interface of titanium. However, the voids are small, but it is the pre-indication point for the crack initiation under fatigue loading. Overall surface morphology of the sample has enhanced grain refinement and is beneficial for the improvement of mechanical properties and wear resistance^39^. Finally, the 3 wt% biochar-included FSW sample was examined, and the micrograph is shown in Fig. 5 (g-h). At this level of concentration, more significant changes were observed on the surface. The high concentration of biochar leads to more particle agglomeration in certain areas and results in larger void formation. Compared to the other samples, the surface irregularities were more, and visible micro-cracks were inspected. Despite this, regions with well-distributed biochar exhibited smoother surfaces, and uniform grain structure was evident from the sample.

### FESEM analysis

**Fig. 6:**
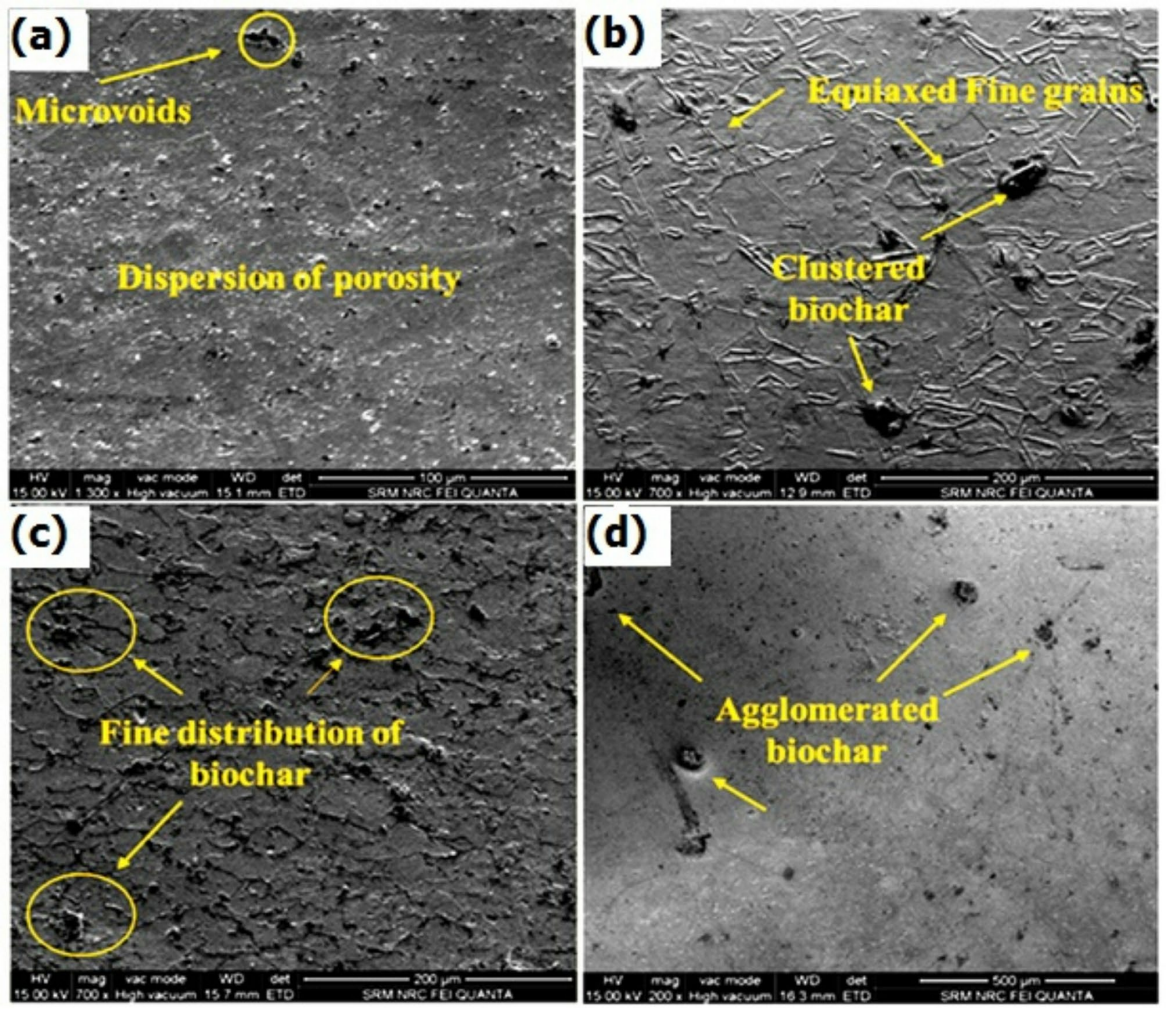
FESEM analysis of (**a**) Plain titanium weld, (**b**) 1 wt% biochar weld, (**c**) 2 wt% biochar weld, and (**d**) 3 wt% biochar weld.

For more detailed morphological analysis, a field emission scanning electron microscope study was performed, and the micrographs are shown in Fig. 6 (a-d). FESEM provides strong details about the particle distribution, interfacial bonding, microstructural changes, and the presence of voids and cracks with higher resolution analysis. The FSW sample without the biochar nanoparticle exhibits an equiaxed grain structure. Under FESEM, the grains are clearer with a well-defined grain boundary network. The evidence of minimal porosities, microvoids, and minor surface asperities was noticed, primarily at the region between TMAZ and the stir zone shown in Fig. 6a. This minor defect results in the generation of stress concentration points and potentially reduces the fatigue resistance under cyclic loading^36,37^. The FSW sample with 1 wt% sample FESEM image was shown in Fig. 6b. Comparatively, biochar was well dispersed throughout the regions and contributing to the grain nucleation. The intense plastic deformation during the FSW process makes this proper grain refinement. From the magnification analysis, the surface appeared relatively smooth with minor surface irregularities. The FESEM images showed a well-blended interface between the titanium matrix and the biochar, enhancing the overall structural integrity of the weld^40^. The analysis of 2 wt% biochar included the FSW sample, and the result was shown in Fig. 6c. From the analysis, a more significant reduction in grain size was observed, and the grains are uniformly distributed. The biochar is well dispersed and leads to the mechanical interlocking and improves the load transfer behavior between the titanium and biochar^40,41^. Also, a few localized forms of biochar clusters were observed, which led to the microvoid formation. The voids formed are scattered with no evidence of larger cracks and defects. The interfaces show good bonding and improve the stability of the weld. Also, the slightly higher surface roughness was noticed with no major irregularities. The grain refinement is carried on the samples by the “pinning” effect mechanism and generates the microstructure with more homogeneous grains. This is because the pinning effect mechanism actively restricts grain boundary movement, leading to grain refinement with a more homogeneous microstructure. Additionally, biochar improves thermal stability by stabilizing grain boundaries at elevated temperatures and prevents excessive grain growth during the welding process^42^.

**Fig. 7:**
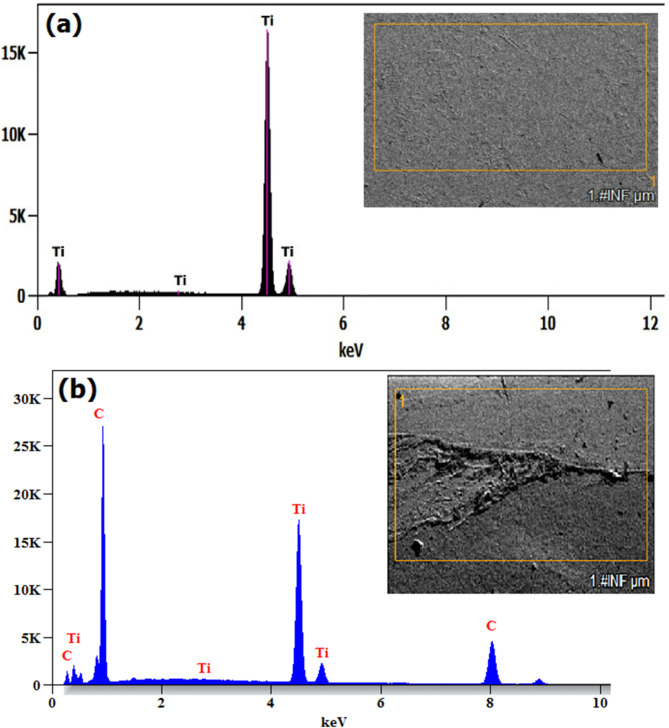
Energy dispersive spectroscopy analysis of (**a**) Plain titanium weld and (**b**) 2 wt% biochar weld joint.

The FSW sample with 3 wt% biochar inclusion sample was morphologically analysed and shown in Fig. 6d. At this concentration level, biochar was more abundant and led to enhanced grain refinement and increased surface defects. At 3 wt%, the higher concentration of biochar increases the dispersion of nanoparticles in the matrix and provides more regions for grain boundary pinning. It results in enhanced grain refinement. But the same higher concentration promotes agglomeration of biochar and generates more localized stress concentrations, potentially compromising weld integrity. However, the grains in the stir zone were finer and indicate the effectiveness of biochar as a grain refiner. Moreover, the evidence of biochar agglomeration was more within the stir zone at specific locations. These clustered particles directed the interfacial cracks and broke the homogeneity of the stir zone and led to increased stress concentration points. The reduced bonding strength at the interface due to the clustering will result in reduced mechanical strength of the weld^8,28^. The specimen prepared was analysed through EDS and verified the presence of biochar. The elemental presence of the reinforcement was analysed through energy dispersive spectroscopy, and these maps are shown in Fig. 7 (a-b). The plain titanium weld was shown in Fig. 7a, which has the absence of carbon material, but sample 3 results precisely ensure the presence of a bulk amount of biochar in the form of the carbon (C) element shown in Fig. 7b.

### Tensile test evaluation

**Fig. 8:**
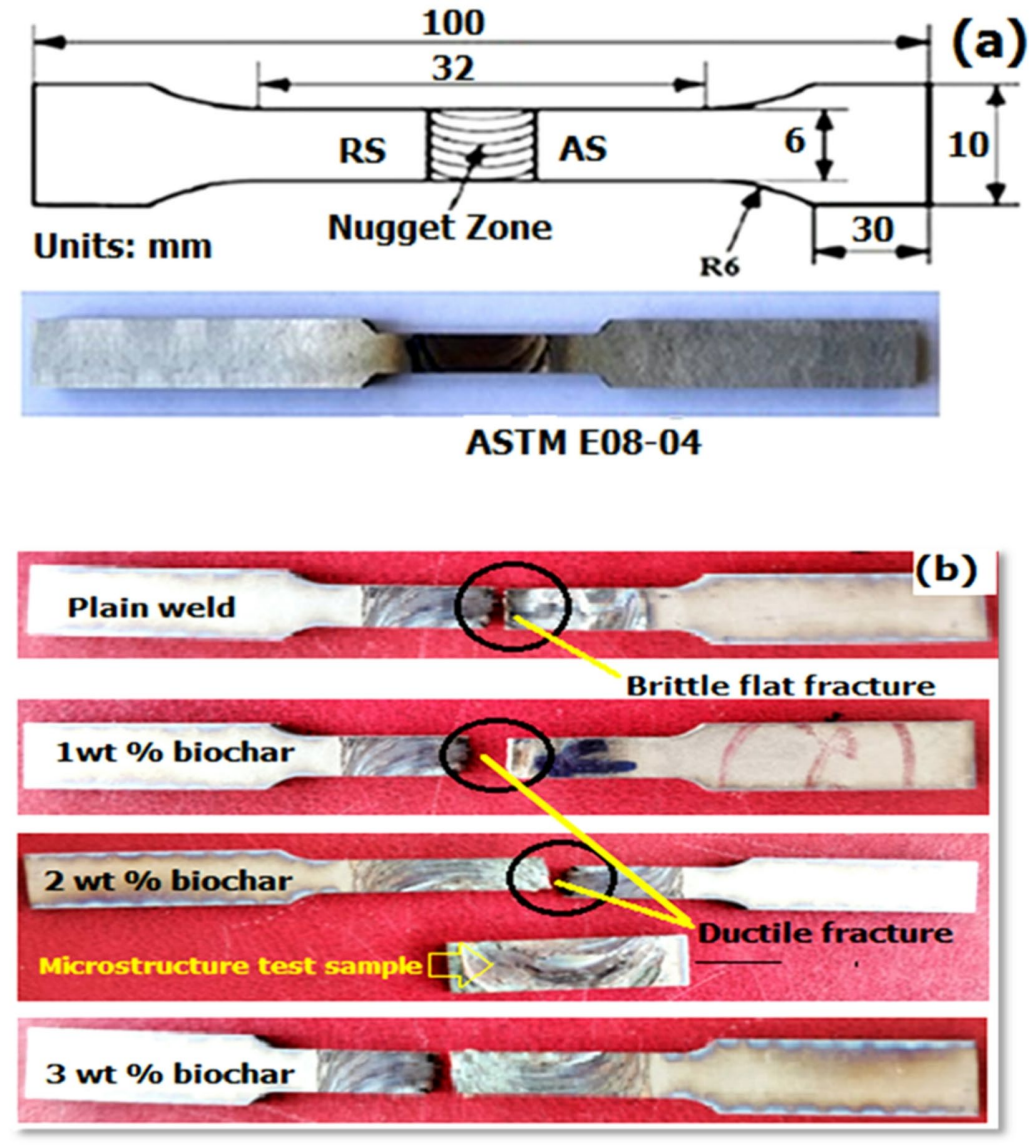
(**a**) Schematic diagram of tensile specimen as per ASTM E08M-04 standard (**b**) Photographic view of fractured tensile test specimens.

The schematic diagram of the tensile specimen with dimensions in accordance with the ASTM E08M-04 standard is illustrated in Fig. 8(a). The tensile testing of the FSW samples with varying biochar nanoparticle inclusions was performed for 3 specimens from each sample, and their average was used for investigation. The fractured tensile test specimens with the addition of biochar vary as shown in Fig. 8(b).

**Fig. 9:**
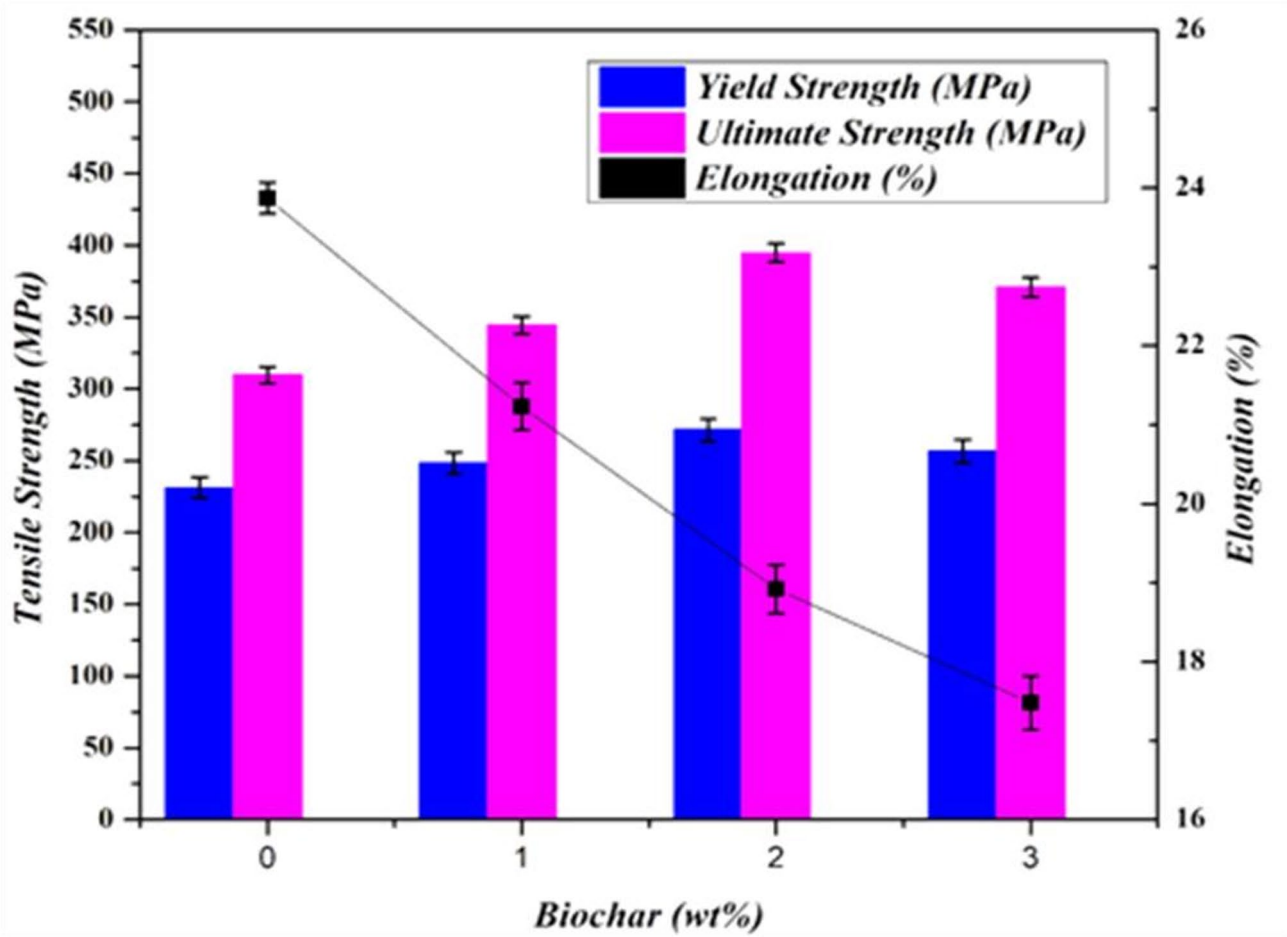
Graphical representation of tensile test results of weld joints.

The objective of the test is to evaluate the influence of processed biochar nanoparticles on titanium welds. The obtained tensile test results of the welded samples are graphically shown in Fig. 9. The FSW sample without biochar inclusion attained moderate tensile strength among the other samples. The stir zone of the weld experiences dynamic recrystallization and contributes to a relatively ductile fracture mechanism with good elongation of 23.9%. However, the absence of biochar limits the dispersion of porosity, leading to the average performance of ultimate and yield tensile strength of 310 MPa and 231 MPa. The addition of 1 wt% biochar-processed FSW sample resulted in moderate improvements in tensile strength due to the even distribution of biochar in the stir zone and grain refinements.

The presence of biochar acts as nucleation sites and increased the load-bearing capacity of the weld^37^. From Fig. 9, the ultimate tensile strength attained was 344 MPa and the yield was 248 MPa, typically more when compared to the reference plain FSW weld sample. However, the reduction in elongation was observed as 21.2%, which was due to the inclusion of biochar, which impedes the material’s ability to plastic deformation. The FSW sample with 2 wt% biochar from Fig. 9 exhibits higher tensile strength results among all tested samples. The ultimate tensile strength increased to 395 MPa, which is a 27.4% improved result compared to the reference plain plate, demonstrating reinforcement effects comparable to those achieved with graphite in polymer matrices^58^. Also, the notable increase in yield strength of 271 MPa was attained. The increased inclusion of biochar promoted grain refinement and led to increased yield and ultimate strength. The biochar is well-dispersed throughout the region and acts as a load-bearing carrier to provide sound resistance to deformation^21,31^. However, the elongation was further reduced to 18.9%, and the level of ductility was still in the acceptable range. From analysing the results (Fig. 9), the 3 wt% biochar FSW sample gets a higher yield (256 MPa) and ultimate strength (371 MPa) than sample 1 and sample 2 but less than sample 3. The reduction in tensile strength was due to the increased inclusion of biochar (3 wt%), which led to particle agglomeration in random areas of the stir zone. This clustering and agglomeration make the formation of localized stress concentrations and microvoids^21,31^. These factors initiate primary fracture during the loading. The elongation of the sample attained was further reduced to 17.5%. The main reason for the reduction in elongation was the clustering of biochar, which acts as a brittle zone within the titanium plates. This makes the initiation of cracks and fractures under tensile stress and results in lower elongation as the material is less able to homogeneously distribute the strain across the weld.

### Fractography analysis

**Fig. 10:**
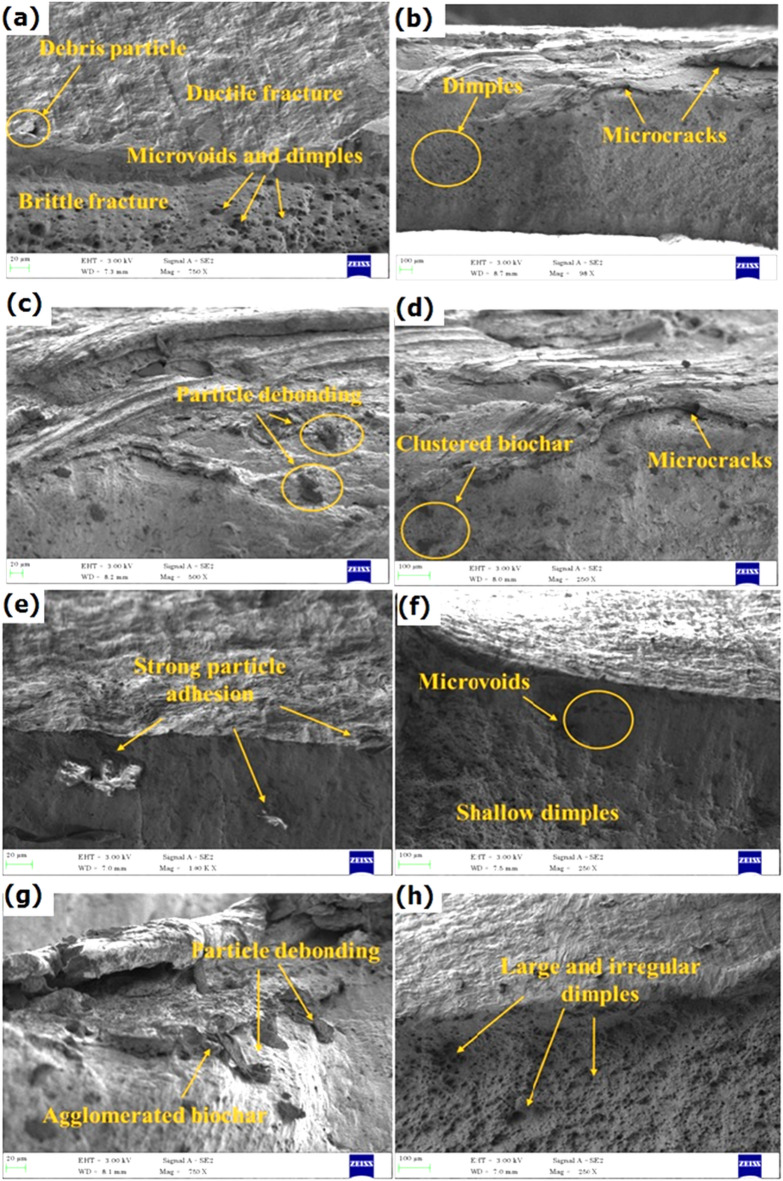
Fractography SEM images of (**a-b**) Plain titanium weld, (**c-d**) 1 wt% biochar weld, (**e-f**) 2 wt% biochar weld, and (**g-h**) 3 wt% biochar weld.

The tensile specimen fractography was analysed through SEM, and the fractographs provide detailed insights into fracture mechanisms, as shown in Fig. 10 (a-h). In the specimen without biochar, the fractured surface has ductile fracture followed by brittle failure. From Fig. 10 (a-b), it was characterized by the indication of deep dimples and fibrous structures. These signs are the plastic deformation of the specimen. The dimples were uniformly distributed and suggest that the strain was significant act before the failure of the specimen during loading. Also, microvoids and microcracks were observed at the boundary regions of the stir zone and TMAZ and act as stress concentration sockets. These defects are responsible for the initiation of cracks and propagate to failure in the stir zone^43,44^.

When analysing the FSW samples with 1 wt% and 2 wt% biochar, finer and shallower dimples on the fracture surface are the signs of enhanced toughness and energy absorption, as shown in Fig. 10 (c-f). The biochar act as reinforcements and increases the strength of the weld. Also, from Fig. 10 (c-f), it was noticed that biochar is well dispersed with strong adhesion bonding and leads to more homogeneous load transfer. This uniformity delays the initiation and propagation of cracks and sustains more load stress. Moreover, Fig. 10 (e-f) shows fewer micro voids with minor signs of particle debonding.

The 3 wt% biochar from Fig. 10 (g-h) showed the presence of agglomerated nanoparticles and larger and irregular dimples with significant voids. The agglomerated and clustered biochar generates weak points in the weld and leads to premature cracks. Also noticed more particle debonding with the titanium matrix. The overabundance of the biochar, which limits the uniformity of the weld and weakens the interfacial bonding^21^.

### Impact test evaluation

**Fig. 11:**
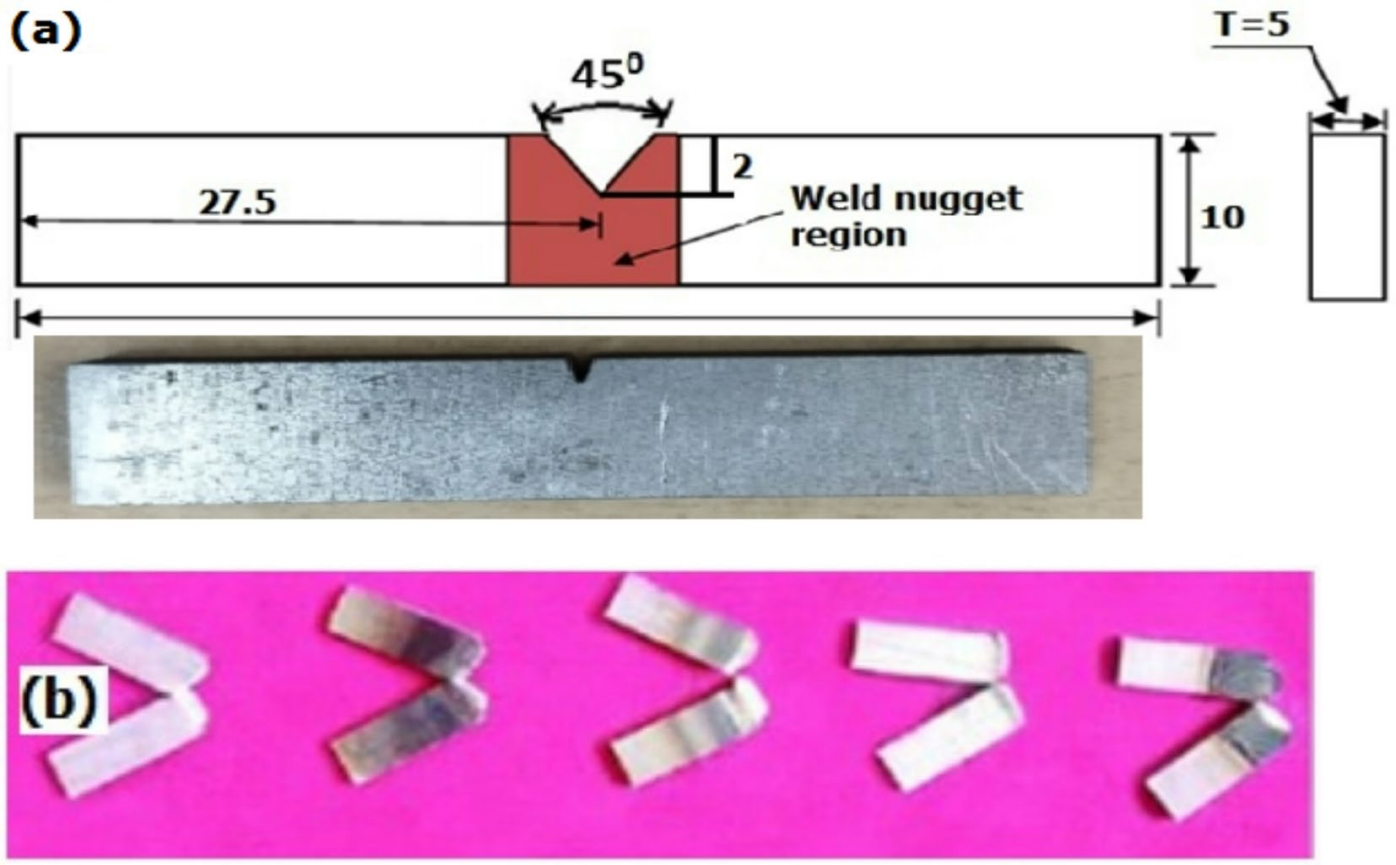
(**a**) Schematic diagram of the Charpy impact test (ASTM E-23). (**b**) Charpy impact test fractured specimens.

The impact toughness of the FSW-processed titanium plate samples was evaluated by Charpy impact tests to investigate the influence of biochar inclusions and their ability to absorb energy during impact. The schematic diagram of the Charpy impact test (ASTM E-23) and tested samples are illustrated in Fig. 11.

**Fig. 12:**
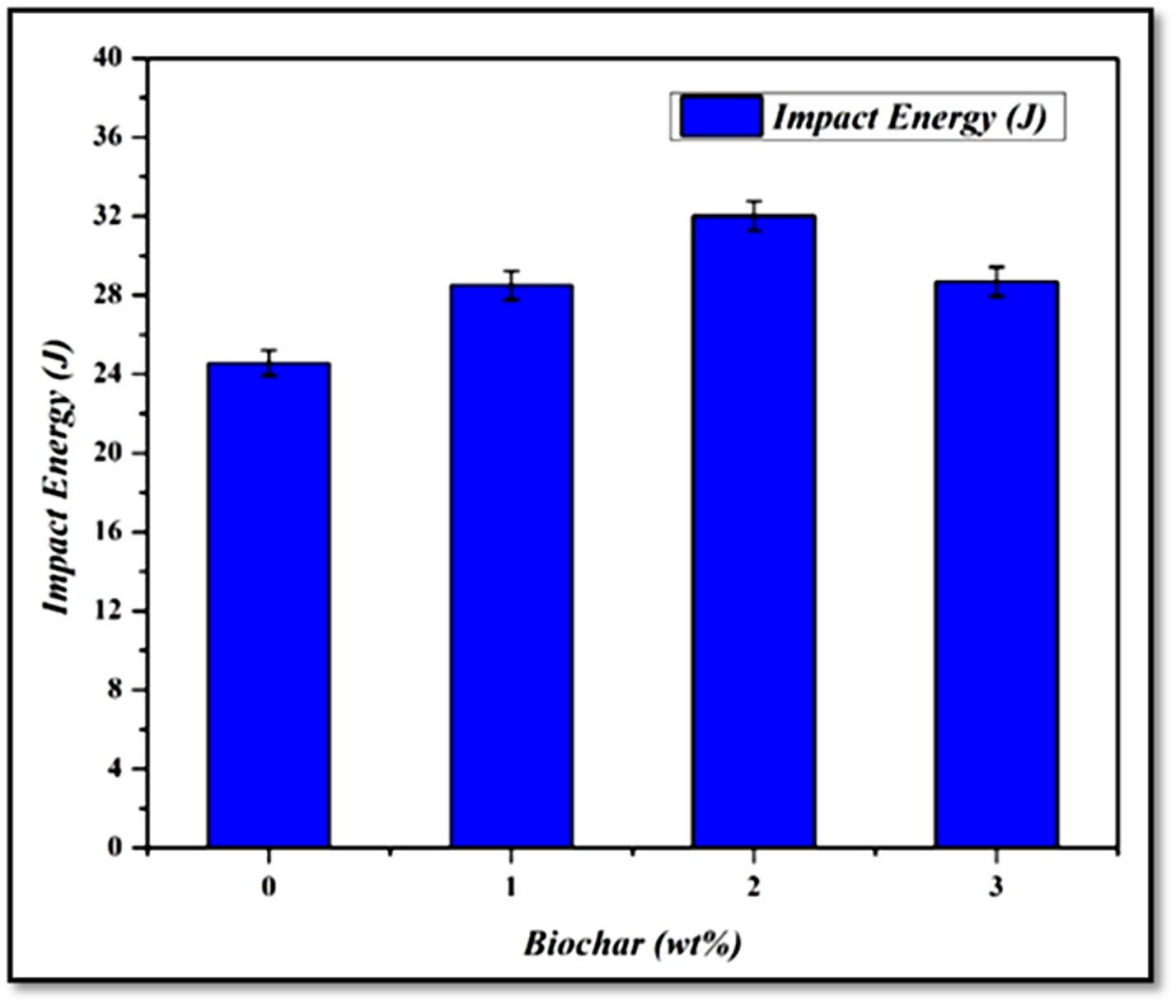
Graphical representation of impact test results of the weld specimens.

The test result was evaluated for three specimens from each sample, and their average was noted for analysis, and the results were graphically shown in Fig. 12. The FSW titanium sample without biochar got the impact result of 24.56 J, and notice that it is the lowest range when compared to the other tested samples. This was mainly due to the absence of biochar limiting the material’s ability to absorb more energy during impact^8^. The inclusion of 1 wt% biochar nanoparticle FSW sample led to the notable improvement in impact result with the energy absorption of 28.5 J, which was a 16.05% increased result compared to the plain plate sample. The added biochar is well distributed in the stir zone and improves the material’s toughness by enhancing the plastic deformation. Also, the biochar improves the grain refinement and limits the formation of crack initiation and propagation during impact load^45^. From Fig. 12, it was analysed that the FSW with 2 wt% biochar inclusion sample exhibits higher impact toughness (32.02 J) compared to the other tested samples. The result attained has a significant improvement of 30.37% over the reference plain weld sample. Biochar acted as a toughening agent when processing with the titanium plates and improved load distribution under impact. The ability of biochar to absorb some of the impact energy and distribute the stresses more evenly contributed to the significant improvement in toughness^21^. The sample with 3 wt% biochar showed a slight reduction in impact result (28.68 J) compared to the 2 wt% sample. Similarly, the increased concentration of biochar makes the onset of particle agglomeration and results in reduced impact toughness. However, the improvement of impact results compared to other samples (1 and 2) was mainly due to the crack deflection mechanism of biochar. Because the increased concentration of biochar acted as a barrier to crack growth during the impact. When a crack was encountered, it was deflected by the biochar, reducing the stress intensity at the crack tip. This crack deflection mechanism allowed the material to absorb more energy before the crack, which propagated through the weld and led to higher toughness^46^.

### Micro hardness evaluation

**Fig. 13:**
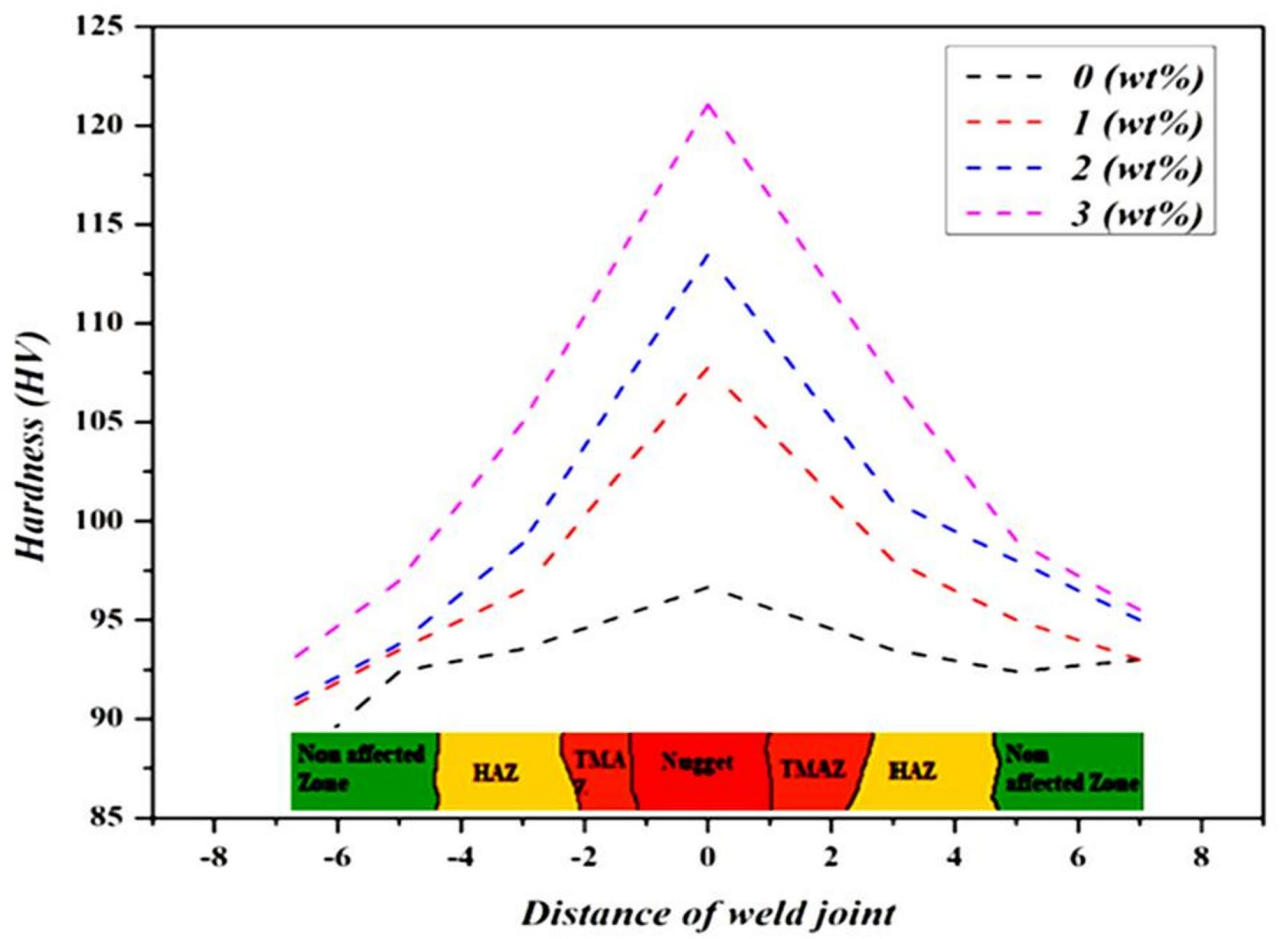
Micro-hardness result of the weld joint.

The hardness characteristics of the weld joints were performed by a Vickers micro-hardness tester, and the results are graphically shown in Fig. 13. The six tests were made on each sample in the HAZ, TMAZ, and weld nugget area, and their average was represented. From analysing the result, it was observed that there was a consistent improvement of hardness with increased biochar content. The FSW titanium plain weld sample exhibits a lower hardness result of 93 HV. This is because the absence of biochar in the stir zone does not provide the hardening effect and results in the lowest hardness value. In comparison, the biochar nanoparticle-included sample gets a notable increase in hardness, the 1 wt% sample attains 98 HV, the 2 wt% sample reaches 101 HV, and the 3 wt% sample records the highest hardness result of 107 HV. The improvement in hardness is mainly due to the effective dispersion of biochar in the weld nugget zone and promotes equiaxed grain refinement in the weld region. As biochar concentration increased, it enhanced the material’s resistance to indentation by impeding dislocation movement, and it is a key mechanism that contributes to hardness^47,48^. Also, the increased concentration of biochar (3 wt%) in the weld zone directly enhanced the load-bearing capacity, making it the most wear-resistant and hardest sample among the tested materials.

### Fatigue test evaluation

**Fig. 14:**
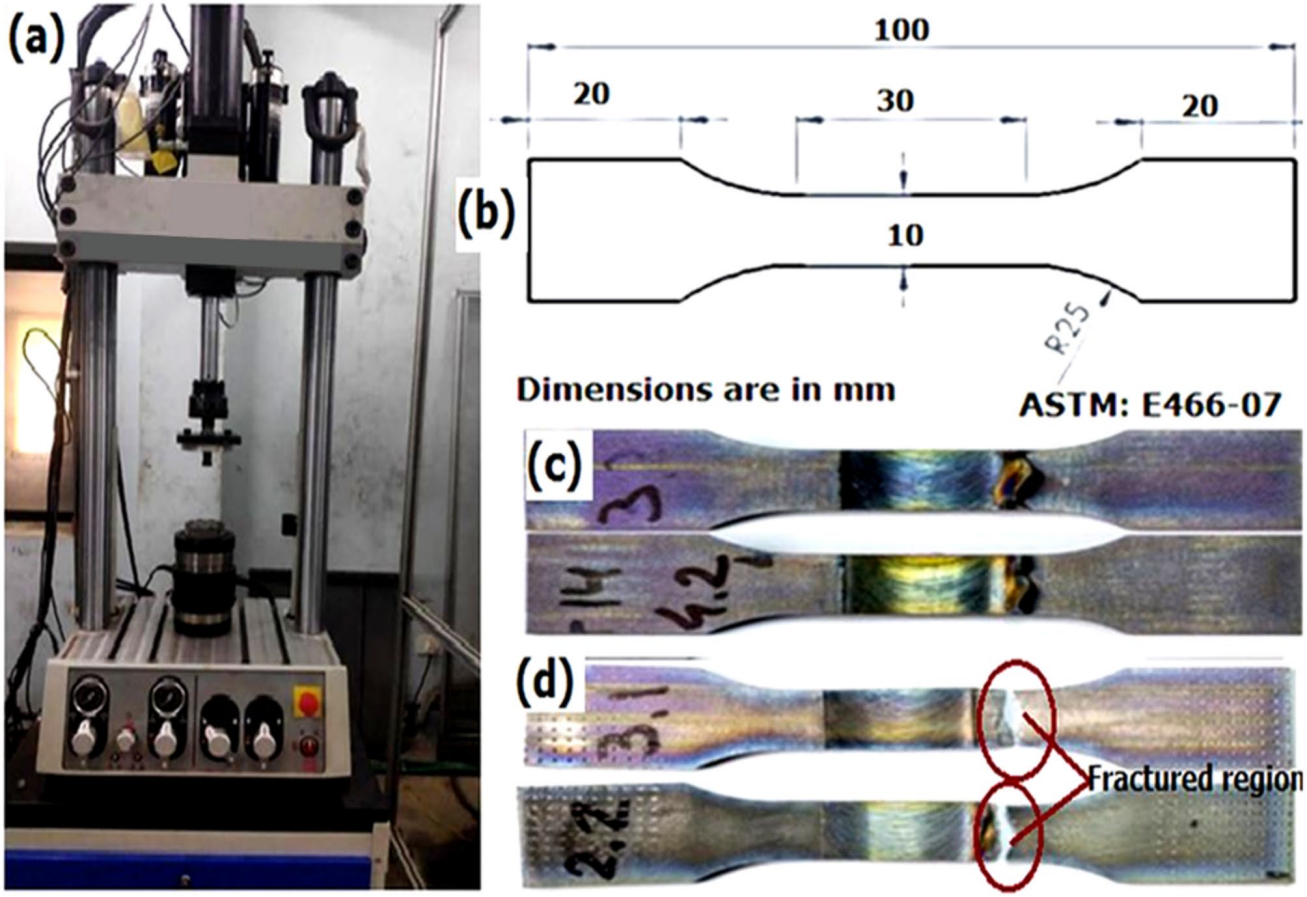
Photographic view of (**a**) Fatigue test machine (**b**) ASTM sample dimensions (**c**) Before (**d**) After fatigue test specimens.

The fatigue strength of various titanium FSW welds prepared with and without biochar reinforcement condition. The photographic view of the fatigue test procedure is shown in Fig. 14 (a-d). It is observed that the bare titanium weld shows a typical nominal fatigue strength of 147 MPa. Due to this lower value, the fine stirring process produces an oxide layer, which causes the heat-affected zones to cool quickly and create high brittle mixtures. The welded area developed tiny equiaxed crystals, while the HAZ and TMT zones saw the formation of new grains of finer size as a result of the fast cooling. Consequently, a lower fatigue strength is measured. Weld zone fatigue strength was immediately enhanced by adding additional small volumes of biochar at concentrations of 0.5, 1, 2, and 3 wt %. The FSW sample without biochar exhibited a result of 147 MPa. The inclusion of a 1 wt% sample attained 165 MPa, which is 12.24% higher than the reference. Continuously, the 2 wt% sample getting higher fatigue strength compared to other samples was 183 MPa and showed a 24.5% enhanced result compared to the reference sample. The fatigue strength was improved due to the effect of biochar nanoparticles, which act as crack deflection and toughening agents, mirroring the stress distribution improvements seen in lightweight composite structures^59^. These factors promote more uniform distribution of stress and delay fatigue crack propagation.

**Fig. 15:**
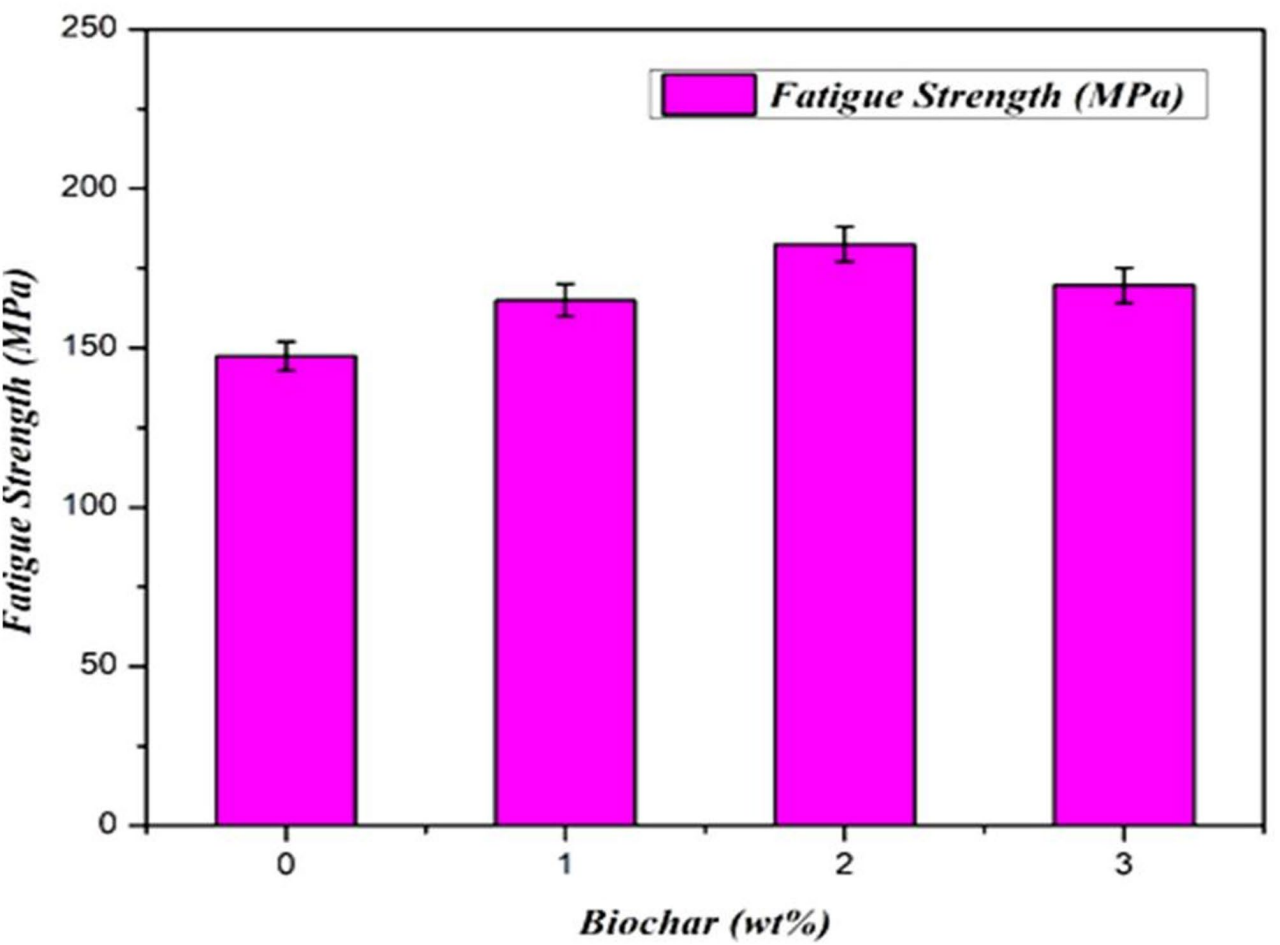
Fatigue test results of the weld joint joints.

The biochar is evenly distributed during the processing, ensuring an even load transfer and enhancing the overall fatigue resistance of the sample. The fatigue strength was slightly reduced for the 3 wt% biochar-included FSW sample, which was 170 MPa, which was a 7% decrease from the 2 wt% sample. This decrease in result is primarily due to particle agglomeration at higher biochar concentrations and generates the localized regions of stress concentration that lead to earlier crack origination and propagation under cyclic loading^49^. But, without the agglomeration issue, the 3% biochar sample still performed better than the 1% sample and indicates that biochar endures to act as a reinforcement even at higher concentrations. The fatigue test results show that FSW plates with biochar nanoparticle inclusion samples get significant improvement in fatigue strength compared to the sample without biochar, and the result was graphically shown in Fig. 15. Also, biochar addition improved resistance by acting as a barrier to crack growth and promoting deflection, which delayed propagation. At 3 wt%, however, particle clustering created localized stress zones, accelerating crack initiation under cyclic loading. The optimum performance at 2 wt% is attributed to uniform particle dispersion, ensuring balanced stress distribution and effective load transfer^50^.

An increase in fatigue strength was achieved in the weld beads by achieving 10^7^ cycles. Because of two different factors, this improvement in fatigue strength has occurred. To begin, the presence of biochar lowers the development of thermally distorted grains by eliminating the heat that is created from the stir zone. As a result, the formation of finer grains is considerably reduced, while the coarse grain size is maintained. A robust lubricating effect is provided by the biochar throughout the welding process. Additionally, the biochar maintains a slow cooling rate, which guarantees that the grains in the TMT and HAZ parts are relatively larger than the grains in the nugget. Second, the effect that carbon has on the weld zone can be described as strengthening. The presence of biocarbon in the stir zone helps to maintain homogeneous load bearing by filling in the spaces that are created as a result of the flow of mixed materials. As a result of the finer carbon, the homogeneity in the weld nugget is improved, which results in a reinforcing effect. This is accomplished by lowering the stress intensity factor in the microscopic surface discontinuities. It has been observed that the significant incorporation of biochar results in a decrease in fatigue strength. Due to the super-saturated mix of titanium intermetallic mix, the fatigue strength is reduced at the marginal level. This is the cause of the drop. The incorporation of additional biochar resulted in the formation of a dense precipitate in the weld zone, which consequently impeded the load-bearing effect and impacted the heat transmission. Additionally, the considerable amount of carbon that is present in the nugget zone causes the weld bead to have a minimal increase in both its hardness and its brittleness. Because of this, the fatigue strength is reported to be slightly lower.

### Wear test evaluation

**Fig. 16:**
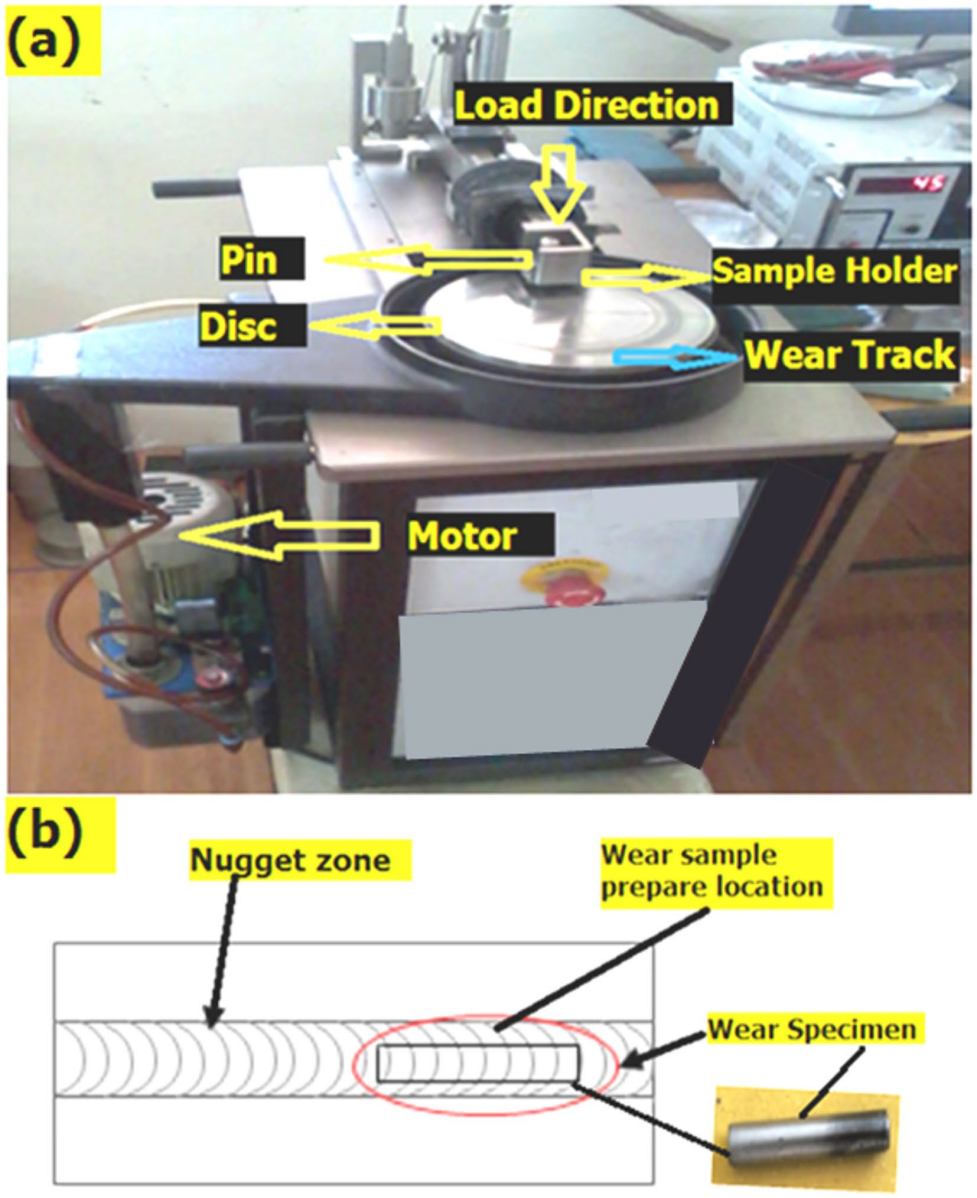
Photographic view of (**a**) Pin-on-disc wear arrangement (**b**) wear Sample & Sample prepared location.

The pin-on-disc tribometer (DUCOM TR-20) was utilized in order to evaluate the dry sliding wear behavior of the friction stir welded samples as shown in Fig. 16. The weld samples were manufactured as pins according to the standard ASTM G99-05. These pins were then tested against the EN 31 hardened steel disc material, which had a hardness level of 62 ± 2 HRC (Rockwell hardness – C). For the purpose of preserving the initial test condition, the pins were rubbed with abrasive paper with grit sizes of 1000 and 1200 simultaneously.

In addition, the face of the disc was scraped with abrasive sandpaper and then wiped with acetone in order to maintain the perfect state after each test. Under the conditions of a constant time period of thirty minutes, a sliding speed of one thousand five hundred revolutions per minute (rpm) was applied, and a weight of ten Newtons was applied. The temperature of the environment was 24℃, with a standard deviation of 20℃. After sliding, the surface of the pin that was being tested was cleaned with tissue paper that had been soaked in acetone, and then it was dried in hot air. The sliding wear loss is determined by calculating the difference between the beginning weight of the pin that was tested and its end weight. The pin that had been made was primarily weighed using an electronic weighing balance that had an accuracy of 0.0001 g. Both the top and bottom sections of each sample were subjected to two separate sets of tests, and the average of those tests was used for the interpretation of the data. For the purpose of measuring sliding wear, the weight loss of the pin was used, and the specific wear rate was determined by employing Eq. 2.

2 $$\:Specific\:wear\:rate\:\left({K}_{s}\right)=\:\frac{\varDelta\:w}{\rho\:D{F}_{n}}$$

where $\:\varDelta\:w$ is the weight loss in ‘gm’, $\:\rho\:$ is the density of the test specimen in ‘gm/cc’, $\:D$ is the sliding distance in ‘m’, and $\:{F}_{n}$ is the applied load in ‘N’.

As the sliding test started between the pin and the disc, the wear and friction monitor continuously showed the frictional force (F_f_) and it was noted to compute the coefficient of friction using the Eq. 3.

3 $$\:\text{C}\text{o}\text{e}\text{f}\text{f}\text{i}\text{c}\text{i}\text{e}\text{n}\text{t}\:\text{o}\text{f}\:\text{f}\text{r}\text{i}\text{c}\text{t}\text{i}\text{o}\text{n}\:\left(\mu\:\right)=\:\frac{Frictional\:force\:\left({F}_{f}\right)}{Applied\:load\:\left({F}_{n}\right)}$$

**Fig. 17:**
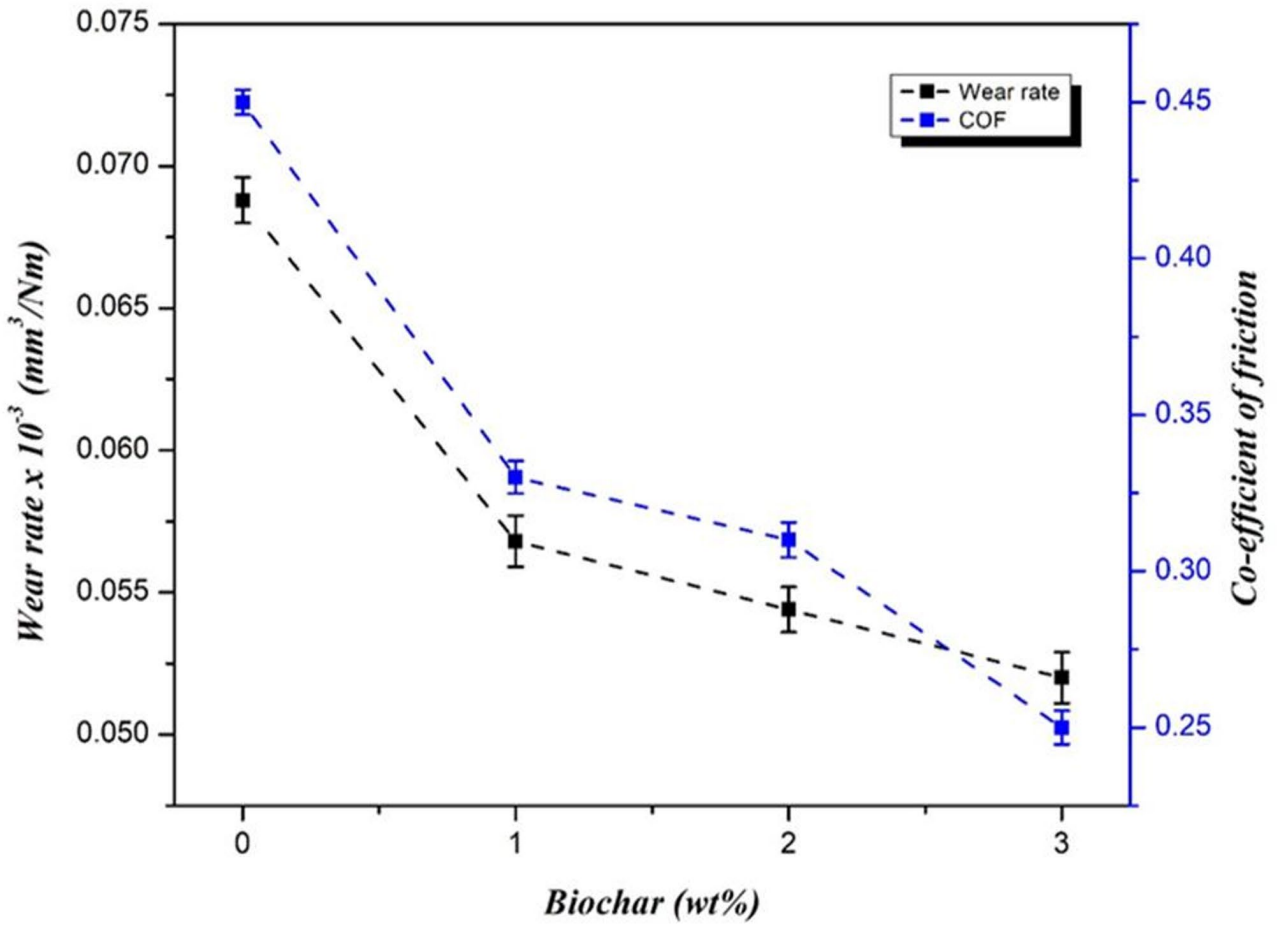
Specific wear rate and co-efficient of friction result of weld.

The dry sliding wear test of FSW titanium plates with different biochar nanoparticle inclusions by pin on disc wear tester by applying the load of 30 N, sliding velocity of 3 m/s, and sliding distance of 1000 m shows a marked improvement in wear resistance compared to the sample without inclusion. The specific wear rate and coefficient of friction results is shown in Fig. 17 as graphical representation. The specific wear rate for the sample 1 (without biochar) was 0.069 × 10^− 3^ mm³/Nm, while the wear rate decreased with the inclusion of biochar. The 1 wt% sample established a specific wear rate of 0.057 × 10^− 3^ mm³/Nm, and the 2 wt% sample further reduced the wear rate to 0.054 × 10^− 3^ mm³/Nm. The best result was observed with the 3 wt% biochar inclusion, which exhibits the lowest specific wear rate of 0.052 × 10^− 3^ mm³/Nm and indicates an extensive improvement in wear resistance. Although the 3 wt% biochar composite exhibited particle agglomeration that adversely affected tensile, impact, and fatigue properties, its wear performance was superior. This is attributed to the higher carbon content, which enhanced surface lubrication and formed a protective tribolayer during sliding. Such mechanisms reduced direct metal to metal contact and minimized material loss. Hence, the wear resistance at 3 wt% biochar balanced the drawbacks observed in other mechanical tests^51^.

The plain FSW sample without biochar had the highest COF of 0.45µ, but the inclusion of biochar has attained reduced friction. The 1 wt% biochar sample exhibited a COF of 0.33 µ, and the 2 wt% sample getting a COF of 0.31 µ, indicates the enhanced sliding wear behavior. The minimum COF of 0.25 µ was observed in the 3 wt% biochar sample, suggesting that the higher biochar content provides the most effective reduction in friction. The main reason for the reduction in COF can be due to the solid lubricating properties of biochar, which reduces the surface adhesion between the sliding pin and disc. The biochar is a carbon-based material with good lubricating properties, likely formed a protective tribolayer during sliding, which minimized direct contact and friction between the surfaces^52^.

The significant improvements in both specific wear rate and coefficient of friction, specifically in the 3 wt% biochar sample. Principally, the dispersion of biochar in the titanium FSW enhances the load-bearing capacity of the material and reduces localized stress concentrations during sliding wear.

The biochar act as barriers to wear and prevents severe material loss and improving the weld hardness. Additionally, excellent thermal stability of biochar helps in dissipating heat generated during sliding and thereby reducing the wear-induced softening of the material. These nanoparticles also aid in developing a tribofilm on the worn surface, which minimizes direct metal-to-metal contact and improves wear resistance. The novel combination of biochar solid lubricating properties and reinforcement mechanisms leads to a significant reduction in wear and friction, making the 3 wt% biochar inclusion the ideal choice for enhancing dry sliding wear performance in FSW titanium plates.

### Worn surface analysis

**Fig. 18:**
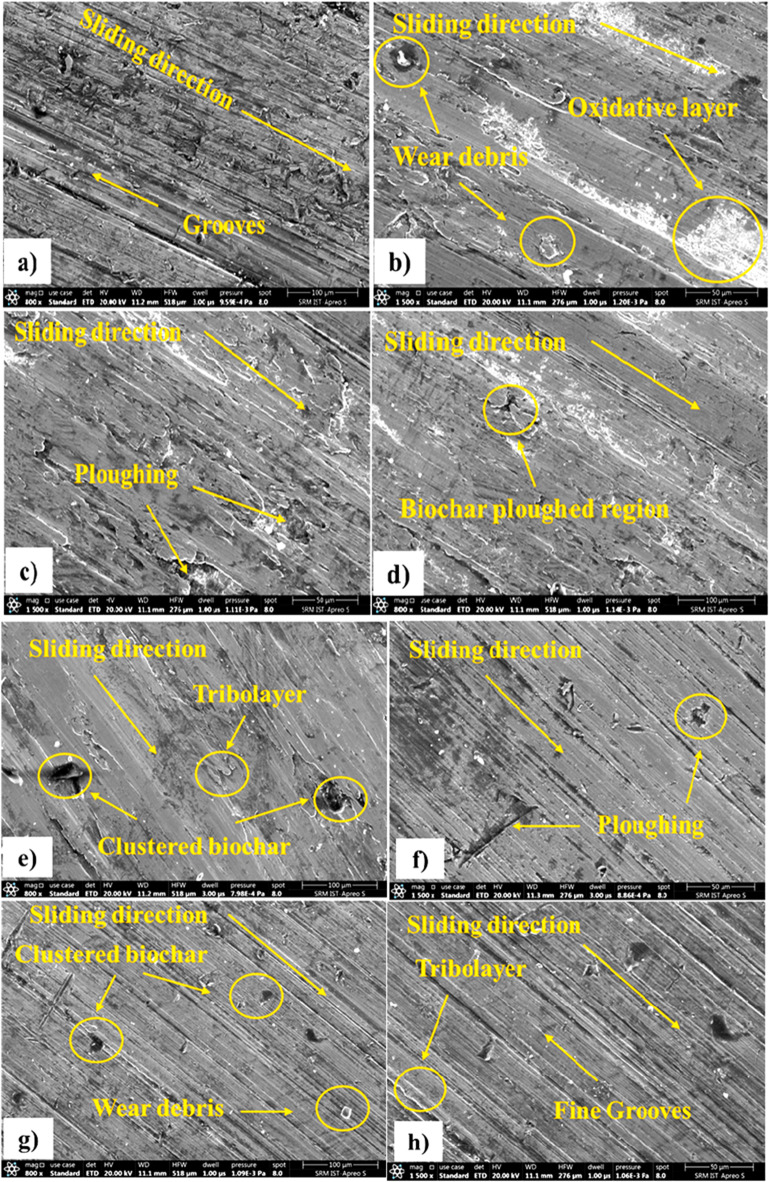
Worn surface analysis of (**a-b**) Plain titanium weld, (**c-d**) 1 wt% biochar weld, (**e-f**) 2 wt% biochar weld, and (**g-h**) 3 wt% biochar weld.

The worn surface analysis by SEM of FSW titanium plates with different biochar inclusions reveals significant differences in wear mechanisms and surface morphology compared to the sample without biochar was shown in Fig. 18 (a-h). In the plain FSW sample, the SEM images from Fig. 18 (a & b) showed deep grooves, severe plastic deformation, and large wear debris, indicating abrasive wear as the dominant mechanism. The material surface exhibited noticeable delamination and signs of adhesive wear, consistent with the high specific wear rate (0.069 mm³/ Nm) and the high coefficient of friction (0.45). These features suggest inadequate resistance to sliding, with significant material loss during wear due to the absence of biochar and mitigate the effects of friction and loads during sliding contact^43,53^.

In contrast, the SEM analysis of the biochar-reinforced samples from Fig. 18 (c & f), particularly the 1% and 2% biochar inclusions, revealed much smoother worn surfaces with finer grooves and less plastic deformation. The 3% biochar sample from Fig. 18 (g & h) showed the most uniform wear pattern, with a protective tribolayer formed on the surface with minimum metal-to-metal direct contact. This tribolayer, likely composed of carbon-based biochar and act as a solid lubricant to reduce both the COF and specific wear rate^54,55^. Also, the Fig. 18 (g-h) shows fewer wear debris and smaller fracture features, indicating that the biochar effectively hindered crack propagation and wear particle formation. The improved surface integrity in the 3 wt% sample highlights the role of biochar in enhancing wear resistance by encouraging a stable and lubricated sliding surface which supports superior dry sliding performance. From the analysis, one of the limitations is agglomeration of biochar at higher concentrations (3 wt%), which lead to localized stress concentrations and uneven wear resistance. Although 3 wt% biochar led to reduced mechanical strength due to particle agglomeration, it showed superior wear resistance. This is due to the formation of a stable tribolayer that minimizes surface damage. The tribolayer acts as a lubricating shield, reducing friction and material loss. Thus, the mechanisms governing wear differ from those affecting strength.

## Conclusion

The present investigation explores the friction stir welding of titanium grade 4 plates with various weight percentages of biochar (0, 1, 2, and 3 wt%) inclusions through various mechanical tests. The quality of the weld and their surface integrity was analysed by microstructural examination. The wear test was made by a pin-on-disc tribometer and their wear behaviour and worn surface analysis were performed. From the detailed investigations, the following conclusions are made.

- The FESEM microstructural analysis revealed that the plain FSW sample without biochar exhibited a grain structure with minimal porosities and microvoids. The samples with biochar inclusion (1, 2, and 3 wt%) have uniform grain distribution, and particularly for the 3 wt% sample, they have particle agglomerations and clustering.
- The tensile test results showed that biochar-induced samples accomplished a higher range of ultimate and yield value. The maximum yield (271 MPa) and ultimate (375 MPa) strength result was found for the 2 wt% biochar-induced FSW sample. But the elongation result was continuously decreased according to the biochar weight%.
- The fractured surface analysis reveals that the addition of biochars improves tensile toughness and energy absorption up to 2 wt% by enhancing dimple uniformity and load distribution, while 3 wt% causes agglomeration, voids, and early fracture due to weakened interfacial bonding.
- The Charpy impact test revealed that the 2 wt% biochar-processed FSW sample had a higher result of 32.02 J.
- The Vickers micro-hardness test shows that 3 wt% biochar induced weld gets maximum hardness result of 101 HV. There was a continuous improvement of results according to the biochar inclusion.
- The 2 wt% biochar FSW sample favors higher fatigue strength of 183 MPa. The 3 wt% sample has a higher fatigue result, but the agglomeration of nanoparticles causes earlier crack origination and failure.
- The dry sliding wear behavior results indicate that the 3 wt% biochar FSW sample achieved a minimum specific wear rate of 0.052 × 10⁻³ mm³/Nm. Additionally, the sample exhibited a coefficient of friction of 0.25 µ.
- The worn surface analysis reveals that biochar-induced FSW samples have smoother worn surfaces with finer grooves and less plastic deformation. The tribolayer was formed on the surface, which is composed of carbon-based biochar and acts as an effective barrier to wear and reduced wear rate and COF.

From the investigations and analysis, the biochar nanoparticle-induced friction welded titanium plate samples have attained improved results in both mechanical and wear behaviour and suggests that they are suitable for the aerospace component applications. The study also encourages the researchers to work on parametric optimization and long-term stability studies to accomplish the precise output.

## Data Availability

The data used to support the findings of this study are included within the article.
